# The ÓMICAS alliance, an international research program on multi-omics for crop breeding optimization

**DOI:** 10.3389/fpls.2022.992663

**Published:** 2022-10-10

**Authors:** Andres Jaramillo-Botero, Julian Colorado, Mauricio Quimbaya, Maria Camila Rebolledo, Mathias Lorieux, Thaura Ghneim-Herrera, Carlos A. Arango, Luis E. Tobón, Jorge Finke, Camilo Rocha, Fernando Muñoz, John J. Riascos, Fernando Silva, Ngonidzashe Chirinda, Mario Caccamo, Klaas Vandepoele, William A. Goddard

**Affiliations:** ^1^ Chemistry and Chemical Engineering Division, California Institute of Technology, Pasadena, CA, United States; ^2^ Optimización Multiescala In-Silico de Cultivos Agrícolas Sostenibles (ÓMICAS) Alliance, Pontificia Universidad Javeriana, Cali, Colombia; ^3^ Facultad de Ingeniería, Departamento de Ingeniería Electrónica, Pontificia Universidad Javeriana, Bogotá, Colombia; ^4^ Facultad de Ingeniería y Ciencias, Departamento de Ciencias Naturales y Matemáticas, Pontificia Universidad Javeriana, Cali, Colombia; ^5^ CIRAD, UMR AGAP, Montpellier, France; ^6^ AGAP, Univ Montpellier, CIRAD, INRA, Montpellier SupAgro, Montpellier, France; ^7^ International Center for Tropical Agriculture (CIAT), Cali, Colombia; ^8^ DIADE, University of Montpellier, CIRAD, IRD, Montpellier, France; ^9^ Facultad de Ciencias Naturales, Departamento de Ciencias Biológicas, Universidad Icesi, Cali, Colombia; ^10^ Facultad de Ciencias Naturales, Departamento de Ciencias Químicas, Universidad Icesi, Cali, Colombia; ^11^ Facultad de Ingeniería y Ciencias, Departamento de Electrónica y Ciencias de la Computación, Pontificia Universidad Javeriana, Cali, Colombia; ^12^ Centro de Investigación de la Caña de Azúcar de Colombia, Centro de Investigación de la Caña de Azúcar (CENICAÑA), Cali, Colombia; ^13^ National Institute of Agricultural Botanics (NIAB), Cambridge, United Kingdom; ^14^ Vlaams Instituut voor Biotechnologie, Bioinformatics Systems Biology, Ghent University, Gent, Belgium

**Keywords:** Multi-omics, crops breeding, foodomics, nanotechnology, rice and sugarcane, in-silico optimization

## Abstract

The OMICAS alliance is part of the Colombian government’s Scientific Ecosystem, established between 2017-2018 to promote world-class research, technological advancement and improved competency of higher education across the nation. Since the program’s kick-off, OMICAS has focused on consolidating and validating a multi-scale, multi-institutional, multi-disciplinary strategy and infrastructure to advance discoveries in plant science and the development of new technological solutions for improving agricultural productivity and sustainability. The strategy and methods described in this article, involve the characterization of different crop models, using high-throughput, real-time phenotyping technologies as well as experimental tissue characterization at different levels of the omics hierarchy and under contrasting conditions, to elucidate epigenome-, genome-, proteome- and metabolome-phenome relationships. The massive data sets are used to derive in-silico models, methods and tools to discover complex underlying structure-function associations, which are then carried over to the production of new germplasm with improved agricultural traits. Here, we describe OMICAS’ R&D trans-disciplinary multi-project architecture, explain the overall strategy and methods for crop-breeding, recent progress and results, and the overarching challenges that lay ahead in the field.

## 1 Introduction

**Figure 1:**
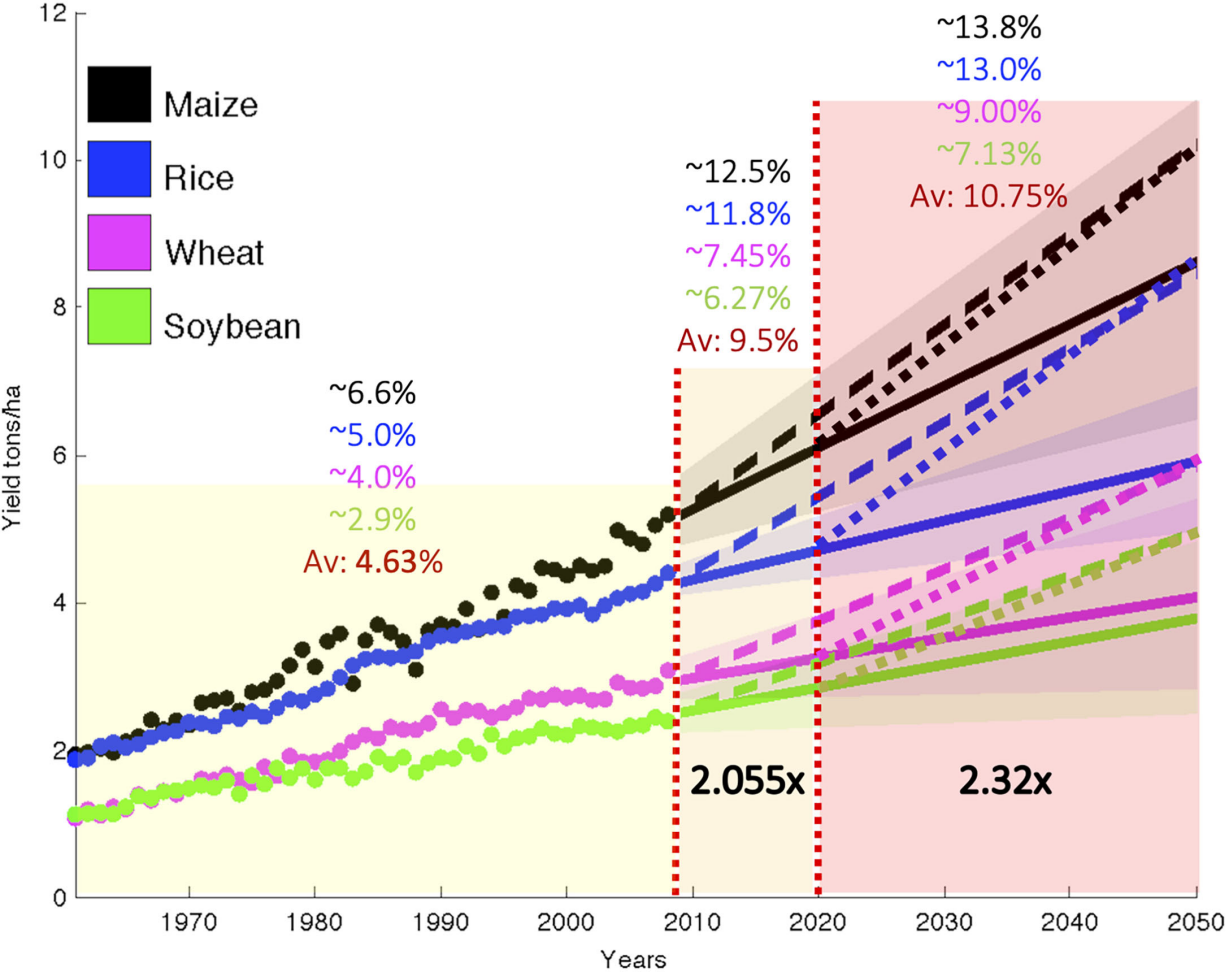
Observed area-weighted global yield 1961–2008 shown using closed circles and projections to 2050 using solid lines for maize, rice, wheat, and soybean. Shading shows the 90% confidence region derived from 99 bootstrapped samples. The dashed line shows the yield trend from 2008 needs to double production, on average, for these crops by 2050, without bringing additional land under cultivation starting in the base year of 2008. The dotted lines from 2020 to 2050 show the yield trend needs to increase by more than 2.3 times with respect to 2008. Adapted from (Ray et al., 2013).

According to the United Nations (UN, 2019), global population will continue to grow throughout the 21st century, to an estimated 10.9 billion by 2100. As a result, food production rates will have to double, which require an unprecedented increase in agricultural productivity, at a rate that has not been seen over the past five decades. **Figure 1** illustrates the scenario for the case of grains, which constitutes more than 40% of the daily protein intake and diet of the global population.

Both biotic and abiotic stresses have altered the production of sustainable crops, in some cases critically. Global food security is permanently challenged by different phenomena including climate change, population growth, human conflict, the reduction of the arable land, and the increased livestock area requirements among several others. From this perspective, it is mandatory for plant breeders worldwide to develop new strategies to deliver crop varieties at a faster rate, i.e., increase the genetic gain for each crop.

Here, we describe the OMICAS alliance and its commitment to the design, development, validation and deployment of an interdisciplinary panomics strategy and tool set to address the sustainability of productive agricultural systems and global food security. OMICAS was selected in 2018, as the sole program in the Food category of the Scientific Colombia ecosystem. Its name, was inspired from the Spanish acronym for *Optimización Multiescala In-Silico de Cultivos Agrícolas Sostenibles* that translates into English as *In-Silico Driven Multiscale Optimization of Sustainable Agricultural Crops*, also corresponds to the suffix ‘omics’ in spanish.

**Figure 2:**
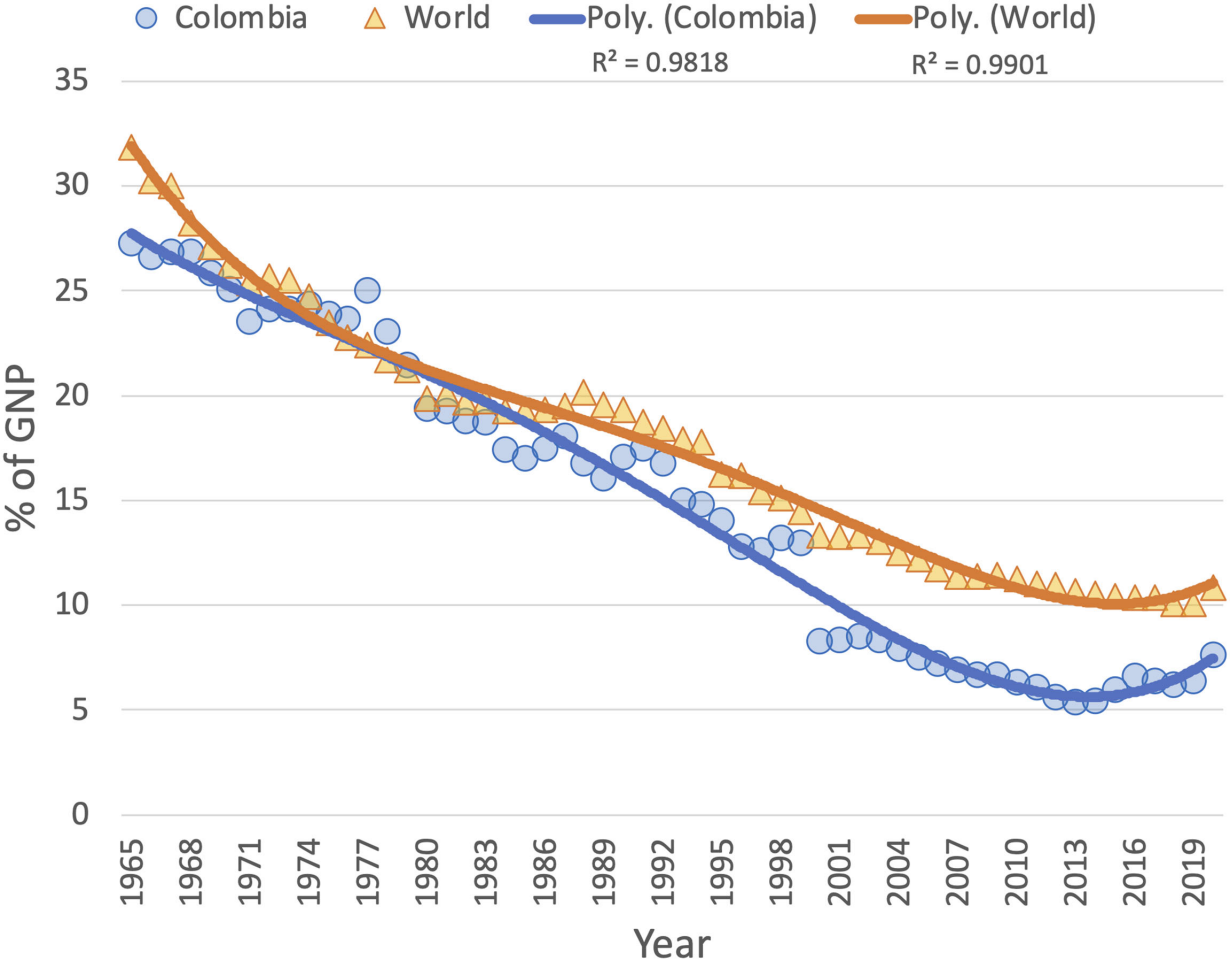
Agricultural sector contribution to Colombia’s and World’s GNP shows a steady decline (faster for the former) in percent contribution since 1965. Includes forestry, hunting and fishing, in addition to growing crops and raising animals. Value added is the net production of the sector, after adding all the products and subtracting the intermediate inputs. It is calculated without making deductions for depreciation of manufactured goods or for depletion and degradation of natural resources. For the countries that count on a value added basis, including Colombia, the gross value added at factor cost is used as the denominator. Source: World Bank National Accounts Data and OECD National Accounts Data Files.

A review from different sources, including the United Nations Development Program (UNDP) (UNDP, 2012), the Food and Agriculture Organization of the United Nations (FAO, 2013), and the Organization for Economic Cooperation and Development (OECD) (OECD, 2015), reveals that over the years Colombia’s agricultural sector has evolved with critical limitations in terms of production, innovation, and technology implementation. In Colombia, agriculture is the primary economic activity of rural territories, and it has experienced multiple structural crisis, which have resulted in a significant reduction of its contribution to the Gross National Product (GNP) from 27% to 5.4% between 1965 and 2013 (**Figure 2**. After hitting an inflection minima in 2013, the sector has shown a recovery in GNP participation up until 2020, when the COVID pandemic hit the world.

In 2017, the Colombian Strategic Plan for Science, Technology, and Innovation of the Agricultural Sector (in Spanish PECTIA) (PEC, 2016) was set in motion in an attempt to consolidate the country’s National System for Agricultural Innovation (SNIA), in Colombia’s post-conflict era. The PECTIA takes into consideration the 3866 Productive Development policy documents from the CONPES (Consejo Nacional de Política Económica y Social, or National Council for Economic and Social Policy) approved in late 2016 (CON, 2015), the recommendations from the Colombian Mission for Rural Transformations, and general orientations provided by the OECD prior to country’s admission as a member in 2018. The PECTIA addresses current societal challenges associated to ‘best-practices’ in agriculture through governance and policy making investment in infrastructure and human resources, as well as financing, planning, tracking, and evaluating strategic projects needed to promote increased productivity and a value-added economy capable of competing in a global market.

The same year PECTIA was unveiled, the Colombian government, through its Ministries of Education, Industry and Tourism, the Colombian Institute of Educational Credit and Technical Studies Abroad (ICETEX), and the Colombian Administrative Department of Science, Technology and Innovation (Colciencias) – now morphed into the Ministry of Science, Technology and Innovation (Minciencias) – created the Colombian Scientific Ecosystem as a two-pronged effort to: 1) promote scientific research and technological development (under the Scientific Colombia program); and 2) graduate-level education abroad (Passport to Science program). Both programs were conceived to prioritize five strategic development areas: Food (Agriculture), Renewable Energy, Health, Society, and Bio-economy, out of which eight international, multi-institutional R&D programs were competitively established. These programs were leveraged by the World Bank through the “Access and Quality in Higher Education Project” (or PACES) program, and anchored at top accredited Colombian Universities.

The application of omics technologies for the improvement of plant traits has enabled significant advances in recent years, as summarized in different reviews; (Großkinsky et al., 2017; Zander et al., 2020; Jamil et al., 2020; Yang et al., 2021) which describe, for the most part, partial integrative approaches, the application of different omics levels to address specific plant models and traits (Rasheed et al., 2013; Singh et al., 2020; Yadav et al., 2022), or the use of modeling and simulation to drive discovery and optimization (Matthews and Marshall-Colón, 2021; Marshall-Colon, 2022). The creation of global networks, such as the International Plant Phenotyping Network (IPPN), (IPPN, 2022) is also contributing to the visibility, information sharing, and application of omics science and technology in agriculture.

OMICAS contributes a unique panomics strategy that couples quantifiable parameters and data, from genome to crop, into functional models for multi-objective optimization of agronomic traits. It not only leverages existing characterization resources, but the development of new sensor and phenotyping technologies for real-time non-invasive characterization of analytes in plants, soils, and atmospheres, and of novel computational methods to elucidate complex inter-omics correlations that become the control knobs to reduce the time and costs in plant breeding. It is a holistic approach, being validated on rice and sugarcane models, whilst extensible to any other crop.

The program brings together leading experts from 17 institutions across the globe, including from four world-class foreign universities (California Institute of Technology, University of Illinois at Urbana Champaign, Ghent University, and Tokyo University), 3 world-class agricultural research institutions (NIAB in Cambridge, UK, the International Center of Tropical Agriculture - CIAT [member of the CGIAR global partnership that unites international organizations engaged in research about food security, located in Colombia], and the Colombian Sugar Cane Research Center - Cenicaña], five major private and public Colombian Universities (Pontificia Universidad Javeriana, Universidad de los Andes, Universidad ICESI, Universidad de Ibague, Universidad del Quindio, and Universidad de los Llanos), and three industrial partners (the Federation of Rice Growers - Fedearroz, Intelecto, and Hi-Tech Automation). The team includes professors, scientific researchers, students and technical staff from a variety of disciplines, including molecular and nano-scale science, ‘omics’ sciences (primarily epigenomics, genomics, transcriptomics, metabolomics, proteomics and phenomics), biology and biotechnology, chemistry, physics, nutrition, computer science, and others, to address the trans-disciplinary challenges associated with sustainable agricultural productivity and food security. This paper presents an outline of OMICAS’ multiscale plant breeding optimization strategy, and describes early results and achievements from the alliance members, primarily validated on two crop models - rice and sugarcane (albeit the strategy, methods and tools are extensible to any other crops). Rice was chosen because it is a major global food source, because it the largest cultivated cereal by surface area in Colombia, and because it has an extensively studied genome. On the other hand, we chose sugarcane, because it is third most cultivated crop by surface area in Colombia, after coffee and oil palm, because it is one of the most efficient plants for photosynthesis, and because it has one of the most complex genomes in crop plants due to the extreme level of polyploidy.

## 2 ÓMICAS R&D architecture

The alliance’s multi-disciplinary research plan involves basic science, as well as the design, implementation, validation, and knowledge transfer in the form of technological solutions aimed at contributing to sustainable agricultural productivity and food security. The main thrusts focus on the omics-level characterization of our two model crops to: establish new breeding strategies, methods, and tools; produce plant varieties with increased tolerance to biotic and abiotic stresses, and with improved resource use efficiency; and reduce the overall environmental footprint of agriculture through updated agronomic practices (specifically greenhouse gas emissions).

**Figure 3:**
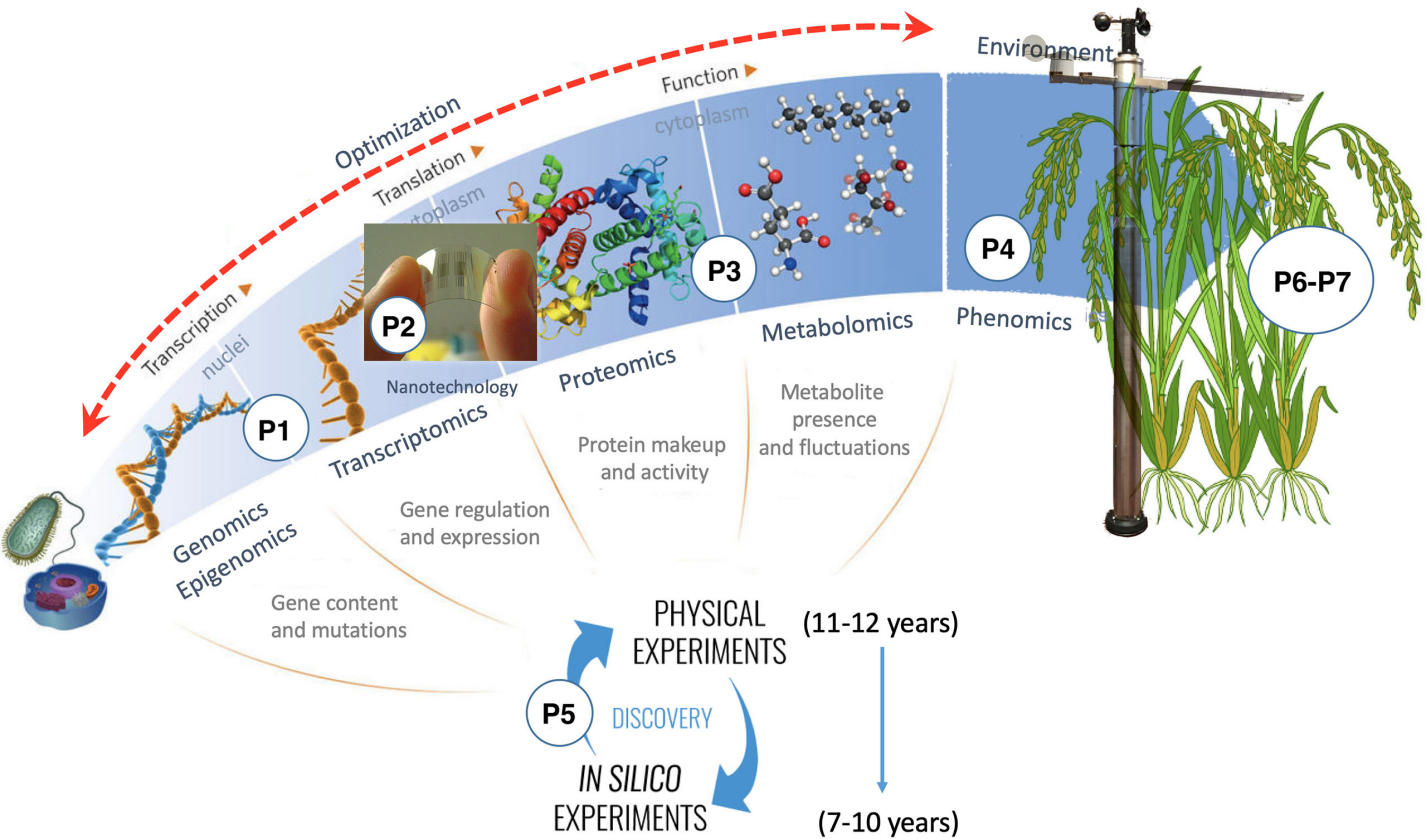
The OMICAS program consists of seven highly-coupled projects, each identified in the figure with a number, the first four build the omics characterization layer of the program, the fifth integrates the characterization data through in-silico models for systematic big data analysis, and projects six and seven use the structure-function relationships obtained from the rest of the projects to develop new varieties with improved traits (validated on rice and sugar-cane models).

**Figure 4:**
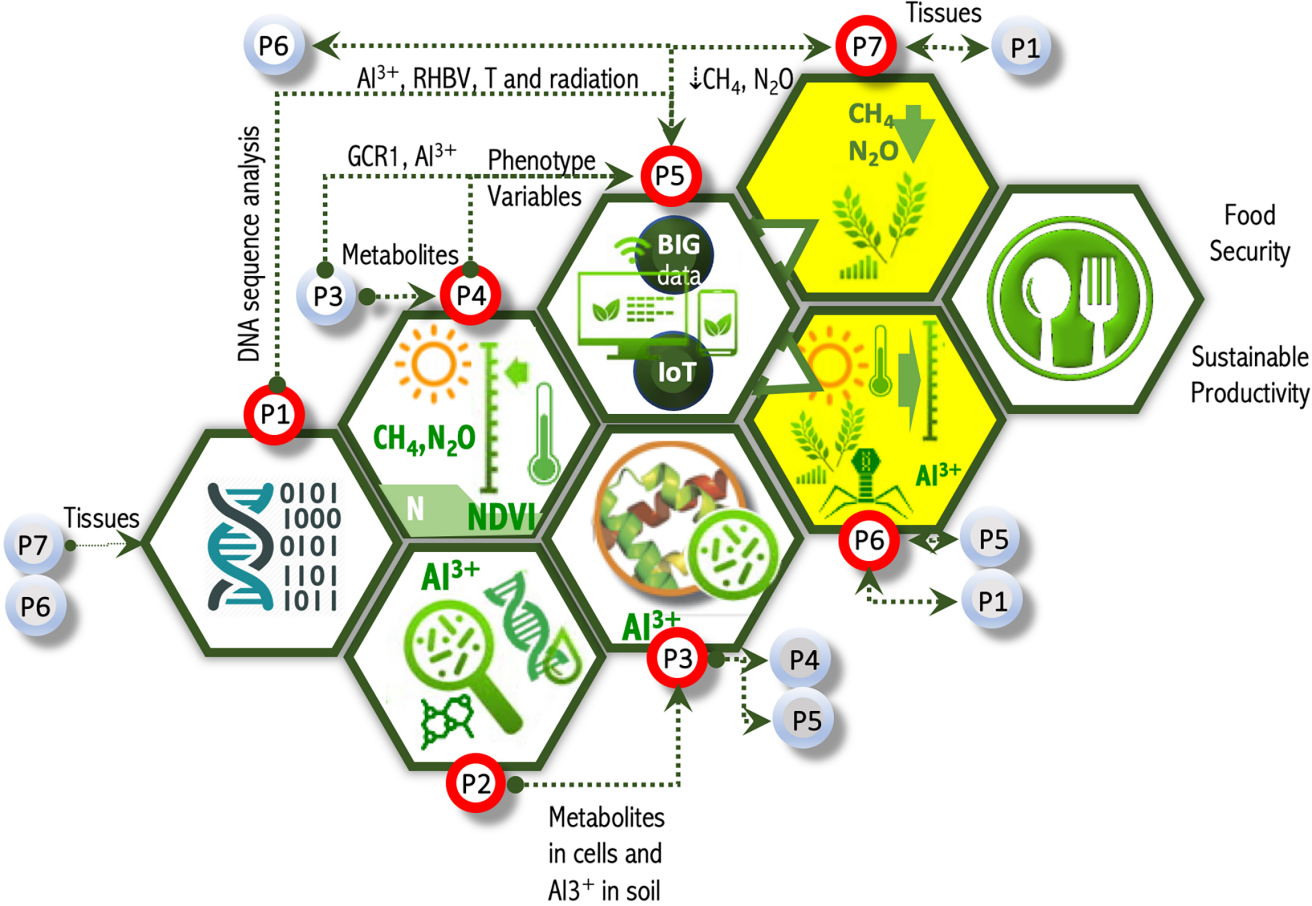
OMICAS program architecture, its macroprojects and their parameter-driven couplings. This figure depicts a sub-set of the basic data couplings in the OMICAS strategy. For example, P1 receives plant tissues from P6 and P7, and outputs DNA/RNA sequences for the modeling efforts in P5; P2 validates sensor technologies on metabolomics and ionomics characterized in P3; P3, in turn, contributes metabolomic and ionomic data for the high-throughput phenotyping effort in P4, and for the development of predictive models in P5; P4 uses molecular and elemental information from P2 and P3, and produces continuum-level phenotypic data for the models and codes developed in P5; while P5, integrates the experimental (physical and computational) multi-omics characterizations from P1-P4 into complex models derived from graph- and network theory, machine learning, and other mathematical and computer science methods to produce predictive tools for in-silico analysis and breeding optimization; P6 and P7 contribute breeding of new plant varieties with improved agronomic traits, based on the in-silico results from P5, and feeds tissues, soil and other environmental samples into the characterization and modeling that occur within P1-P5.

OMICAS is composed of seven interrelated macro projects, identified in **Figure 3** as P1 through P7 and coupled as shown in **Figure 4**. These projects contribute to the overarching goals of the program, as follows:

1. Development and implementation of an experimental and computational platform for genomic, transcriptomic, and epigenomic plant processing and analysis, and of bioinformatic tools for the analysis and integration of molecular scale data associated with crop productivity,
2. Design, characterization, and fabrication of prototype nanodevices for the detection and measurement of ultra low-concentrations of tissue biomarkers (specifically, primary and secondary metabolites, and aluminum metal *Al* ^3+^ ions in soils), in order to enable early, fast, and high-resolution identification of plant response to stress,
3. Profiling of metabolic pathways for simple sugars, organic acids, phenolics, flavonoids, and dextrans in crops, using targeted and non-targeted metabolomic methods and the high-resolution phenotyping technologies derived from P1, and elucidation of key cell signaling mechanisms from plant cell-membrane receptors, specifically GCR1, to establish their role in stress response,
4. Development and implementation of an integrated low-cost, high-throughput, geographically-distributed multimodal phenotyping platform (fixed, mobile and aerial) that integrates soil-plant-atmosphere variables during meristematic, elongation, and maturation phases of plant growth,
5. Development of computational models and data visualization tools for in-silico analysis and optimization of crops, based on graph theory and big data analytics algorithms, aimed at gene annotation, identification of gene and metabolic circuits associated with productivity and tolerance to stress conditions.
6. Applying the methods and tools developed by P1-P5 to the identification and annotation of genes, the development and selection of promising germplasm, and the design of new plant varieties with greater performance in productivity and stability in the presence of diseases (e.g. rice hoja blanca virus), climate changes (i.e. low or high temperatures and radiation), heavy metal soil toxicity (e.g. *Al* ^3+^), and optimal resource use efficiency (e.g. non-structural carbohydrates).
7. Applying the methods and tools developed by P1-P5 to identify and select plant varieties with reduced greenhouse gas emissions (specifically N_2_O and CH_4_) that favor soil conservation and minimal environmental footprint.

This alliance is committed to contributing basic, measurable, and transferable solutions to these problems, including but not limited to: new omics characterization and analysis techniques and tools, candidate genome sequences, candidate quantitative trait locus (QTLs) and genes, and optimized germplasm. A key element in OMICAS is the integration of an in-silico and physical experimental optimization cycle, based on epigenomic, genomic, metabolomic, and proteomic data and its correlation with phenomic expression to enable elucidation of complex genotype-phenotype relationships. The in-silico components are meant to improve breeding throughput, and to reduce the cost and time involved in traditional methods. The ‘omic’ characterization layer allows for multiobjective optimization of agricultural traits, such as, resource use efficiency, nutrient sink-source translocation efficiency, resistance to different biotic and abiotic stresses, and minimization of the environmental footprint. This multiscale characterization approach is essential to elucidate molecular-level structure-function relationships that affect gene expression, metabolic regulation, and an organism’s response to its environment.

## 3 Results and discussion

The use of whole-genome data, derived from high-throughput sequencing technologies, in association with accurate crop phenotyping, has allowed the discovery of genetic traits that control phenotypic variations in crops.

### 3.1 Epigenetic and genetic characterization of crops

In P1, we have advanced in the implementation of an experimental and computational platform for storage, processing, analysis, and biological interpretation of epigenetic (methylation profiles) and genetic crop data. We have established an epigenomic analysis strategy supported by computational models and experimental methods to characterize yield and differential responses to biotic and abiotic factors in the target crops of rice and sugar-cane. Furthermore, we are developing and validating novel bioinformatics strategies and flows for analysis and visualization of structural and functional genomics.

The focus is placed on the dynamic epigenetic processes that modulate access to DNA in response to upstream signals including DNA methylation, covalent modification of histones, nucleosome remodeling, chromatin interaction with regulatory long noncoding RNAs. These are critical to ultimately understand gene expression.

P1 has established an experimental platform supported by the implementation of computational tools for the analysis of massive omics characterization data. The project integrates a physical layer for handling and processing experimental tissue samples, and a complementary computational high-performance computing (HPC) infrastructure (a GPGPU cluster set up at the alliance’s anchor institution) for the storage and analysis of omics data generated. This data will be released to the public domain as the infrastructure grows. Three major computational-experimental efforts are under way between P1 and other projects in OMICAS, one (with P6) meant to identify epigenetic cues associated to reducing the effect of abiotic stresses (specific case of *Al* ^3+^ toxicity from acid soils), a second (again with P6) meant to uncover the genotypic and phenotypic variations underlying sucrose production, and a third (with P5) meant to systematically annotate genes from genome-phenome data using machine learning methods.

**Figure 5:**
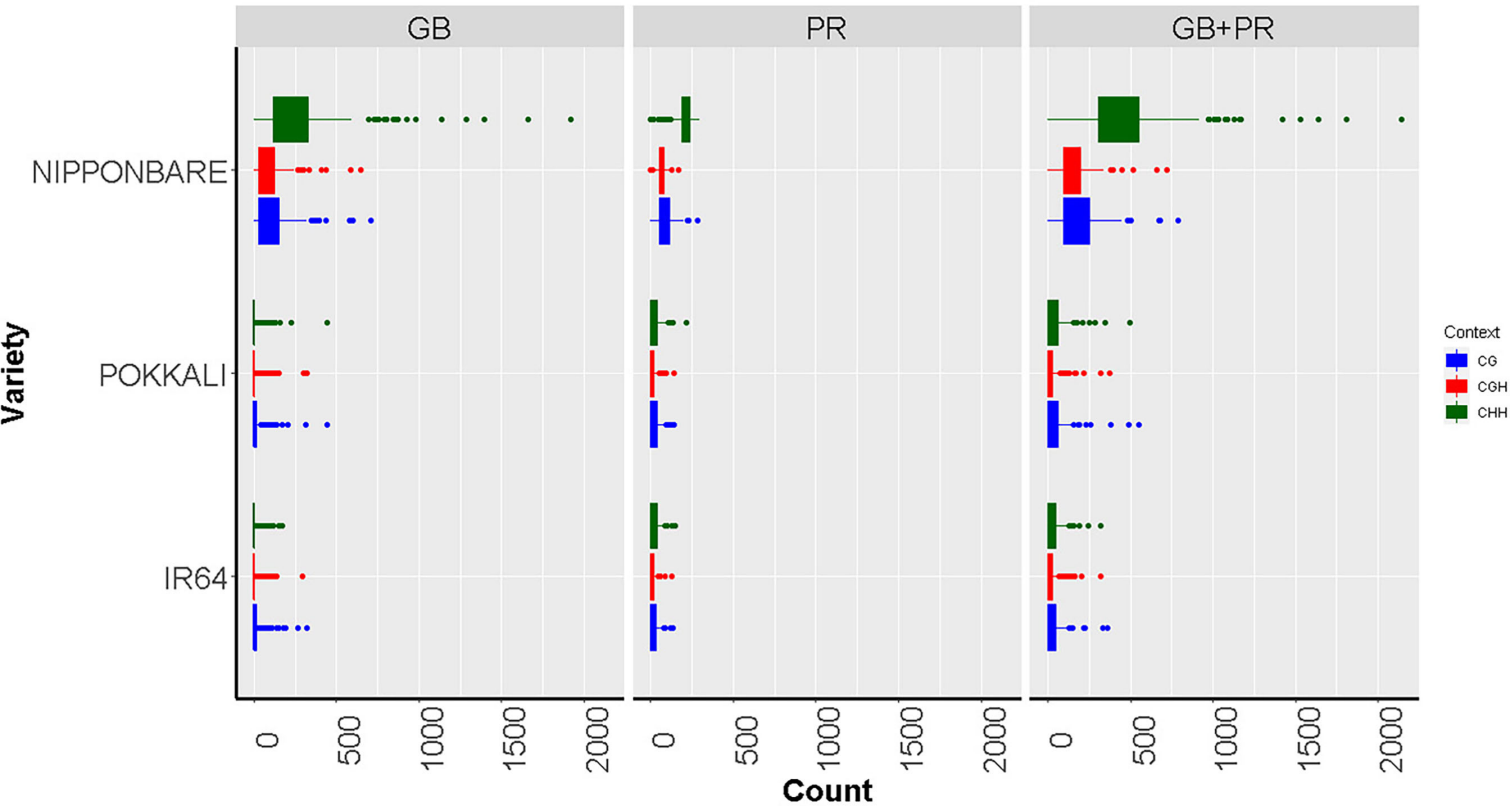
Boxplots showing methylated cytosine frequency in three sequence contexts: CG (blue), CHG (red), and CHH (green) among three different rice varieties with contrast responses to aluminum exposure: Nipponbare (Tolerant), Pokkali, and IR64 (Susceptible). The results are discriminated according to the location of the epigenetic mark, either inside the gene body region (GB), the promoter (PR), or both the promoter and inside the gene body region of analyzed genes (PR + GB). From (Gallo-Franco et al., 2020).

For the first case, we are progressing in an epigenomic study to characterize the methylation patterns in four commercial rice cultivars (*Oryza sativa L.* and two accessions of wild rice (*Oryza glumaepatula Steud*, through whole genome bisulfite sequencing. Differential epigenetic marks will be evaluated between rice genotypes with a contrasting response to aluminum stress under controlled conditions. By using this strategy, epigenetic changes will be considered as fixed epigenetic marks. Likewise, the changes in the methylation patterns between the aluminum tolerant and susceptible rice genotypes will be evaluated after being subjected to *Al* ^3+^ toxicity conditions, and the epigenetic changes identified will be considered rapid epigenetic marks in response to aluminum stress. Once the specific differential methylation patterns have been identified, expression levels of genes that had been found to be differentially methylated, between tolerant and susceptible genotypes, will be evaluated by qPCR. With all the epigenetic and transcriptional information, functional enrichment analyzes will be carried and a functional response model to aluminum stress will be developed. This will represent a significant advance in understanding the epigenetic mechanisms in the response to abiotic stresses in plants, in particular to understand the key mechanism in the regulatory response of rice crops to aluminum toxicity. This information will be transferred to different breeding programs worldwide. Our early findings, based on methylation analysis from Nipponbare cultivar (highly tolerant to Al toxicity) and IR64 and Pokkali varieties (susceptible to Al toxicity) indicate that Nipponbare exhibited more methylated sites than the other two varieties (p≤0.01 in an FDR analysis), while IR64 and Pokkali did not show differences in methylation - see **Figure 5**. These results are particularly interesting, given Nipponbare has been extensively reported as a highly tolerant cultivar to aluminum (Gallo-Franco et al., 2020).

For the second case there were two approaches implemented to identify variations underlying sucrose production. The first one consists of performing the identification of molecular DNA markers throughout the implementation of a genome-wide association analysis (GWAS). To do so, a core collection of 220 sugarcane genotypes, which covers the genetic and commercial diversity from Cenicaña’s germplasm bank, were phenotyped during two crop cycles at a field representative from the humid environment of the valle del río Cauca, Colombia. Similarly, each one of the 220 genotypes were sequenced with a high-throughput whole genome sequencing strategy, in order to massively identify Single Nucleotide Polymorphisms (SNPs). Finally, both phenotypic and genotypic information were combined through the QK-mixed linear model (Yu et al., 2006) to find SNPs associated with sucrose production. Preliminary findings indicates the presence of 28 SNPs associated with sucrose content at 13 months after planting, from which only 4 explains between 5 and 10% (*R*_2_>5%) of the total phenotypic variation observed in the 220 genotypes. These results suggest that sucruse production is a quatitative trait that is highly influenced by environmental effects, with several minor QTLs that control its production. To validate the association of each one of the 28 SNPs, we planted a population of 150 sugarcane genotypes, with sucrose production between 5 and 18% in a humid environment. This population will be phenotyped for sucrose production and, at the same time, sequenced with targeted sequencing technologies to look for the allele dossage for each SNP and to confirm the overall impact on sucrose production. The second approach, consists of the quantitative evaluation of the analyzed genotypes by means of the multiscale phenotyping strategy in OMICAS. This involved identifying a set of 4 genotypes with more than 16% sucrose-producing accessions, as high-producing, and with less than 7%, as low-producing. These genotypes were planted in fields from a sugarcane mill in the Valle del Cauca, in Colombia. We will now perform an epigenetic study aimed at finding epi-alleles that could assist the breeding scheme at Cenicaña. Therefore, at harvest time (around 13 months after planting), tissue from the low and high-sucrose-producing genotypes will be collected and sequenced through whole genome bisulfite sequencing. Finally, differentially expressed markers will be evaluated against sucrose production and considered as fixed epigenetic marks or epi-alleles. In this way, not only the genome structural variation will be taken into account to establish direct genotype-phenotype associations with evaluated traits, but also, significant differential epigenetic marks will corroborate and help us elucidate those defined associations.

A major challenge in agriculture is incorporating genomic information into functional plant breeding. A holistic approach is mandatory to directly apply genomics-derived knowledge into agronomy, both at the molecular (genomic through metabolomic) and macroscopic (phenotypic) levels, and for deriving a plant’s response under contrasting conditions (i.e. normal and stressed). With this goal in mind, we are performing specific phenotype-genotype associations for different agronomic traits, and have developed strategies for the analysis and integration of complex data using comparative genomics approaches, bioinformatics and big data analysis tools. This will generate new pipelines for our model crops and for others. For example, we have now developed a new method for in-silico prediction of functional gene annotations in rice. This approach uses gene annotations from existing knowledge of a given genome in combination with topological properties of its gene co-expression network, to train a supervised machine learning model that is designed to discover unknown annotations. The approach was validated to functionally annotate the Oryza Sativa Japonica genome. It uses any existing body of knowledge about gene annotations for a given genome, and the topological properties of its gene co-expression network, to train a supervised machine learning model that is designed to discover unknown annotations. These results, sumarized in **Table 1**, revealed that the topological properties derived from co-expression networks improve our predictions for annotating genes (Romero et al., 2020).

**Table 1:** Number of genes most frequently annotated as false positives for the 32 annotations by our model, trained from topological metrics extracted from an Oryza Sativa Japonica genome.

ID	Biological process	# Genes	Max FP	# FP
0006807	Nitrogen compound metabolic process	15	41	1
0006289	Nucleotide-excision repair	20	46	1
0006397	mRNA processing	17	48	1
0007017	Microtubule-based process	18	49	1
0070588	Calcium ion transmembrane transport	10	36	1
0006184	GTP catabolic process	49	47	1
0044267	Cellular protein metabolic process	25	49	1
0007186	G-protein coupled receptor protein signaling	11	50	1
0006281	DNA repair	62	50	2
0006754	ATP biosynthetic process	24	49	3
0006904	Vesicle docking involved in exocytosis	11	50	4
0055114	Oxidation-reduction process	870	47	5
0006886	Intracellular protein transport	135	50	19
0006855	Drug transmembrane transport	32	50	21
0006662	Glycerol ether metabolic process	28	50	27
0006888	ER to Golgi vesicle-mediated transport	16	50	29
0006259	DNA metabolic process	15	50	32
0007067	Mitosis	11	48	33
0008652	Cellular amino acid biosynthetic process	18	50	52
0030244	Cellulose biosynthetic process	23	50	64
0034968	Histone lysine methylation	11	50	93
0006812	Cation transport	62	50	96
0045454	Cell redox homeostasis	83	49	103
0006506	GPI anchor biosynthetic process	12	50	284
0007165	Signal transduction	104	50	370
0071805	Potassium ion transmembrane transport	24	50	570
0006357	Regulation of transcription from RNA polymera	12	50	1199
0006396	RNA processing	58	50	1212
0044237	Cellular metabolic process	75	50	1318
0006457	Protein folding	162	50	2358
0006952	Defense response	133	50	2679
0006096	Glycolysis	50	50	2875

The ‘Max FP’ column summarizes the number of times (out of a total of 50) such an annotation is suggested for a gene, while the ‘FP’ column identifies the number of genes that are consistently given such an annotation. From (Romero et al., 2020).

We expect that the combined use of traditional genomic and epigenomic characterization strategies, coupled with the use of novel techniques based on holistic analysis, will lead the identification of novel gene/molecular mechanisms aimed at reducing the times to develop agronomically improved varieties.

### 3.2 Characterization of plant biomarkers

In P2 we are developing nanoscale sensors for the detection and measurement of bio-markers (primary and secondary metabolites, including non-structural carbohydrates and organic acids) in plants and toxins in soils (*Al* ^3+^). A plant’s response to biotic and abiotic stresses has an early molecular-level expression in the organism’s metabolome, which is prior to any phenotypic variations, that can signal proliferation of diseases, compromised productivity, etc. Metabolites provide a direct window into the phenotype, to the physiological state of the plant. These fuel cell signaling and regulate metabolic activity in the plant, so characterizing and associating their concentrations in time with cellular processes, can further our understanding of genome-phenome relationships.

Identifying metabolite-mediated signaling pathways, in real-time, *in-vivo*, selectively (targeted metabolomics), at ultra-low concentrations (pico Moles, pM, or lower), cheaply and without harming the host organism is not only of fundamental importance, but a practical necessity for agriculture. Unfortunately, current technologies for measuring metabolites, such as nuclear magnetic resonance spectroscopy (NMR), high-performance liquid chromatography (HPLC), alone or in tandem with mass spectrometry (HPLC-MS), inductively coupled plasma mass spectrometry (ICP-MS), and enzyme-based methods, fall short of meeting these needs. These lack portability, and tend to be expensive to acquire and operate.

Phenotypic changes in response to biotic and abiotic stresses are reflected early on in an organism’s metabolome, hence the need to measure key metabolites for improving early detection of stresses and breeding stress-tolerant species. In our design process, we include both first-principles based in silico screening and experimental prototyping. Our focus is placed on three different sensing platforms:

- Electronic field effect devices (FED): Back-gated transistor devices that translate electronic field effect variations proportional to an analyte’s concentration on a functionalized semiconducting channel’s surface into changes in transconductance/voltage/current across two or more terminals,
- Colorimetric/optical devices or assays (OD): Functionalized metal nanoparticle systems that fluoresce under UV excitation to produce an intensity signal response proportional, or inversely proportional, to an analyte’s concentration in solution, and
- Electrochemical devices (ECD): Functionalized nano-structured electrodes that produce distinguishable voltammetric, impedanciometric or amperometric signals as a function of an analyte’s concentration on the electrode’s surface (electrochemical sensors).

**Figure 6:**
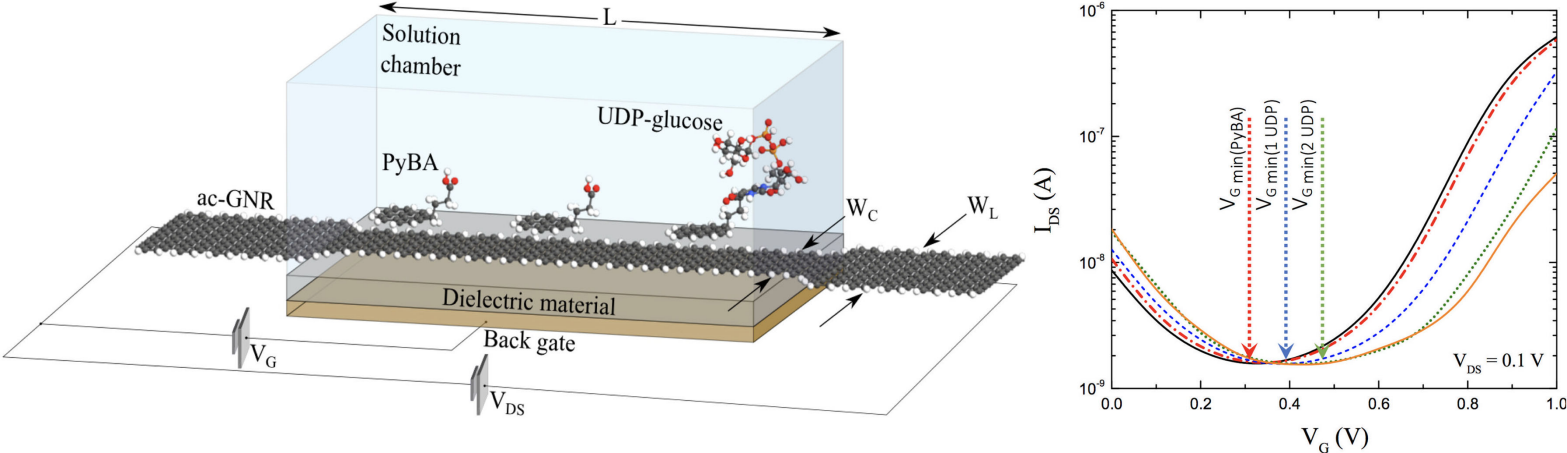
(Left) Isometric view of graphene-based FED sensor with solvent box, 3 PyBA SAM molecules, and 1 bound UDP-glucose molecule. A metallic back-gate, with an 8 nm thick separating region with relative dielectric constant of 3.9 (i.e. *SiO*2), lay under the semiconducting junction; (right) Transconductance shows *p*-doping effect of increasing UDP-glucose concentration (at *V_DS_* =0.1 V). Figures from (Jaramillo-Botero and Marmolejo-Tejada, 2019).

In our FED designs, the semiconducting channel surface is modified with molecular receptors that are selective to the analyte of interest. The attachment of target analytes to the receptors, result in the depletion or accumulation of charge carriers in the semiconducting channel, analogous to the effect of a transistor base/gate terminal. In (Jaramillo-Botero and Marmolejo-Tejada, 2019), Jaramillo-Botero and Marmolejo demonstrated a low-voltage solution- and back–gated graphene nanoribbon (GNR) field–effect transistor (GFET) sensor design, for the detection and measurement of low-concentration (pM) uridine diphosphate glucose (UDP-glucose), a precursor to sucrose synthesis in a plant cell’s cytoplasm and an extracellular signaling molecule capable of activating downstream defense mechanisms, see **Figure 6**. A self-assembled monolayer (SAM) of 1-pyrenebutyric acid (PyBA) was used to noncovalently functionalize the graphene surface on one end, and to covalently ligate UDP-glucose on its open end, whilst providing mechanical, chemical and electronic signal sensing stability. The device has a predicted limit of detection (LOD) of *0.997/n* mM/L (where *n* is the number of sensor units in an array configuration), with high transconductance sensitivity, 0.75-1.5 *μ*S for 1-3 UDP-glucose molecules, at low input (*V*_G_=0.9V) and output voltages *V*_DS_=0.1V. Thus, a 1000x1000 nanoarray sensor would yield a LOD of *0.997* nM/L. See **Figure 6**. This low-power, all-armchair g-FET sensor with SAM ligands that may be chosen to bind different biomarkers, provides a unique opportunity for high throughput, real-time, low-cost, high-mobility, and minimal-calibration sensing applications for in-field phenotyping.

Nanoparticle-based fluorescent probes offer an alternative solution to quantify plant analytes directly from exudates or by direct absorption into the tissues. Nanoparticles (NPs) with the proper size (<100nm), composition and surface modifications can be absorbed onto the cell membrane and subsequently internalized into the cytoplasm. Detailed information about the analyte’s concentration can be retrieved wirelessly, by modifying the NP’s surface with analyte-selective moieties and small molecular weight fluorophores/chromophores to signal the presence or absence of targeted analytes on these sites. The use of NPs has the added benefit of increasing the total surface area available for binding analytes, when compared to a flat electrode surface. We demonstrated a fluorophore-functionalized gold nanoparticles (AuNP) systems for colorimetric detection and quantification of sucrose and other plant analytes as described in (Jaramillo-Botero et al., 2019). Absorption of radiation (typically in the UV spectrum, i.e. relatively high *hv*) promotes an electron from its electronic ground state to an excited state. During the lifetime of the excited electronic state, part of the energy is lost through internal molecular vibration, leading to a longer wavelength of the released/emitted light (Stokes shift). When the fluorophores emit part of this light as radiation, the AuNPs act as a collisional quenchers of the excited state thereby reducing the fluorescence intensity. The fluorophore then returns to the ground state without light emission. The fluorescence wavelength and distribution of the emitting fluorophore is chosen to overlap the absorption spectra of the AuNP, and the length (R_1_) of the mercapto-oligomers that connect the fluorophore to the AuNP is chosen to maximize quenching at such a distance. The analyte concentration is therefore inversely proportional to a differential fluorescent signal, with respect to the amount of fluorophores content.

Last, but not least, we have developed disposable carbon-based electrochemical sensors for the detection and quantification of different metabolites in plants, metals in soils, and greenhouse gases. These can be used in the field with a portable instrument or as part of a phenotyping platform, in real-time, and with minimal cost. We are now able to selectively quantify the presence of Al^3+^ ions in dry and acidic soils, as an indicator of its bioavailability. We expect to use the same technology to quantify it in different tissues, in order to study and understand its effect on plant metabolism. Aluminum ion uptake impairs synthesis, cell expansion, and nutrient transfer from plant roots to main stems, affecting their overall metabolism (Barceló and Poschenrieder, 1990). In Camila Ayala et al. (2022), we demonstrate and validated a glassy carbon electrode modified by the electrochemical reduction of bismuth in an acetate buffer, for the detection of Al^3+^ in a cupferron solution, using double-potential pulse chrono-amperometry. The sensor has a linear response in the concentration range of 1.85x10^10^ to 3.70x10^6^ mol/L and a detection limit of 0.025ppb. Our current technology, uses laser-scribed graphene electrodes, which enable scaling production and tuning the sensor’s sensitivity range.

In general, nanostructured electrodes or assays can provide the resolution and accuracy required for detecting and quantifying ultra-low analyte concentrations, from samples captured *via* iontophoresis, natural exudation or gutation processes directly from a plant’s leaf, stem or root. Sensors can be tattooed onto the plant surface of interest, in a ‘wearable’ device configuration, or they can be embedded into other fixed or mobile instruments. These technologies are amenable to industrial scaling and production and are key to improving agroindustrial productivity and safety.

### 3.3 Characterizing stress signaling through membrane protein complexes

In P3 we are studying G-protein signaling in plants, using a combination of first-principles based membrane protein-structure prediction and experiments on mutants. Stress signalling across the cell membrane remains a fundamental biological question in plant science. Although G protein-coupled receptor (GPCR) analogs in plants have not yet been conclusively determined, we believe G proteins transmit signals by atypical mechanisms in plants (when compared to humans and animals) while effector proteins control growth, cell proliferation, defense, stomatal movements, channel regulation, sugar sensing and some hormone-mediated responses, as shown by Murano et al (Urano et al., 2013) using *Arabidopsis thaliana* and rice (Oryza sativa) models. Genome analysis identified 56 putative GPCRs, including G protein-coupled receptor1 (GCR1), which is reportedly a remote homologue to human class A, B, and E GPCRs (Taddese et al., 2014). Taddesse et al (Taddese et al., 2014). addressed the disparity between genome analysis and biological evidence through a structural bioinformatics study, involving fold recognition methods, from which only GCR1 emerged as a strong candidate. The activation of GPCR analogs in plants defines their function, and it involves multiple distinct conformations that do not follow in step with animal G signalling, as described by Apone et al (Apone et al., 2003). Moreover, since some G protein components are capable of activating more than one intracellular (IC) signaling pathway, it is essential to identify the multiple active conformations that may be involved with different functions.

**Figure 7:**
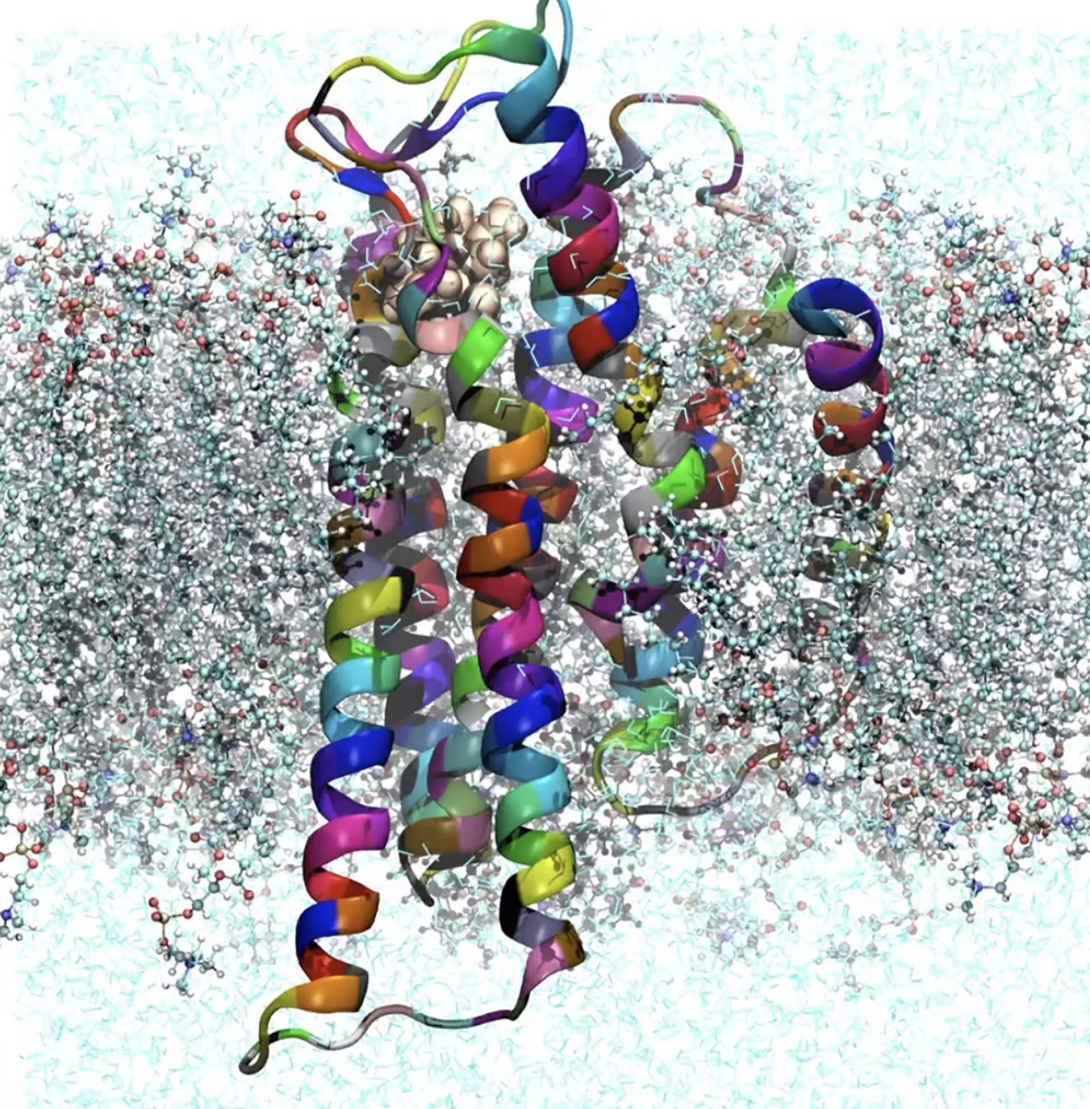
Predicted GCR1 3D atomic structure and composition embedded in a bi-lipid cell membrane and in explicit water. The structure was obtained from the primary of *Arabidopsis Thaliana* using a hybrid approach based on homology and our first-principles methods (Hernandez et al. (2022).

To understand the GCR1 activation mechanisms using modeling, accurate three-dimensional (3D) structures are required. However, these are not currently available from crystallographic or NMR experiments, therefore we are leveraging on the first-principles based approach from Goddard et al (Vaidehi et al., 2002; Goddard et al., 2010) to predict and validate the tertiary GCR1 structure from its primary sequence. The predicted structure (see **Figure 7** are used in nano-to-micro second molecular dynamics (MD) simulations to determine the potential activation mechanisms and signalling pathways. We are currently supplementing Simulation results using stress-response characterization of *Arabidopsis thaliana* ecotypes and knock-out mutants, and performing gene annotation and analysis to determine stress responses, before moving to a functional validation of a high-performing rice haploid (haplotypes) for particular agronomical traits of interest.

As depicted in **Figure 4**, P3 is also focused on the characterization of non-structural carbohydrates (NSC), secondary metabolites (e.g. flavonoids), and *Al* ^3+^ ions in acid soils. One major objective was to identify the role of NSC (specifically, sucrose and starch) and secondary metabolites (i.e., phenols and flavonoids) as signaling elements that regulate a plant’s performance under biotic and abiotic stresses. On the other hand, toxicity from *Al* ^3+^ affects the absorption ofessential nutrients (such as *Ca*_2+_) and restricts the normal growth of its roots. This alters essential physiological processes of a plant, and quenches plant productivity. The phytotoxic effects of aluminium are highly-dependent on the concentration of *Al* ^3+^ and the plant’s genotype ability to translocate the metal from source to sink. With P2, we have now demonstrated a rapid, real-time electrochemical sensor for measuring ppm-levels of *Al* ^3+^ in soils; a tool that will undoubtedly contribute to the development of plant varieties with improved tolerance to metal toxicity, accurate selection of crops for acid soils, and to soil remediation strategies (Ayala et al., 2022).

### 3.4 Phenotypic characterization of crops

Increased productivity in agriculture will lead to greater availability and lower costs for both food and non-food products derived from agronomic practices. The efficiency of resource allocation among farmers needs to be characterized through sustainable agriculture. High-performance phenotyping (HTP) strategies and platforms are necessary to optimize the acquisition of data from individual plants and large crop plots. These techniques allow farmers and breeders to access real time data on the status of their crops, improved crop management, and proper selection and optimization of species as a function of microclimate and soil conditions. This involves new sensing technologies capable of resolutions beyond the continuous variables at the macroscopic scale, down to the level of molecules, integrated within low-cost, low-power, massively distributed HTP and new ontologies to facilitate data integration and analysis.

**Figure 8:**
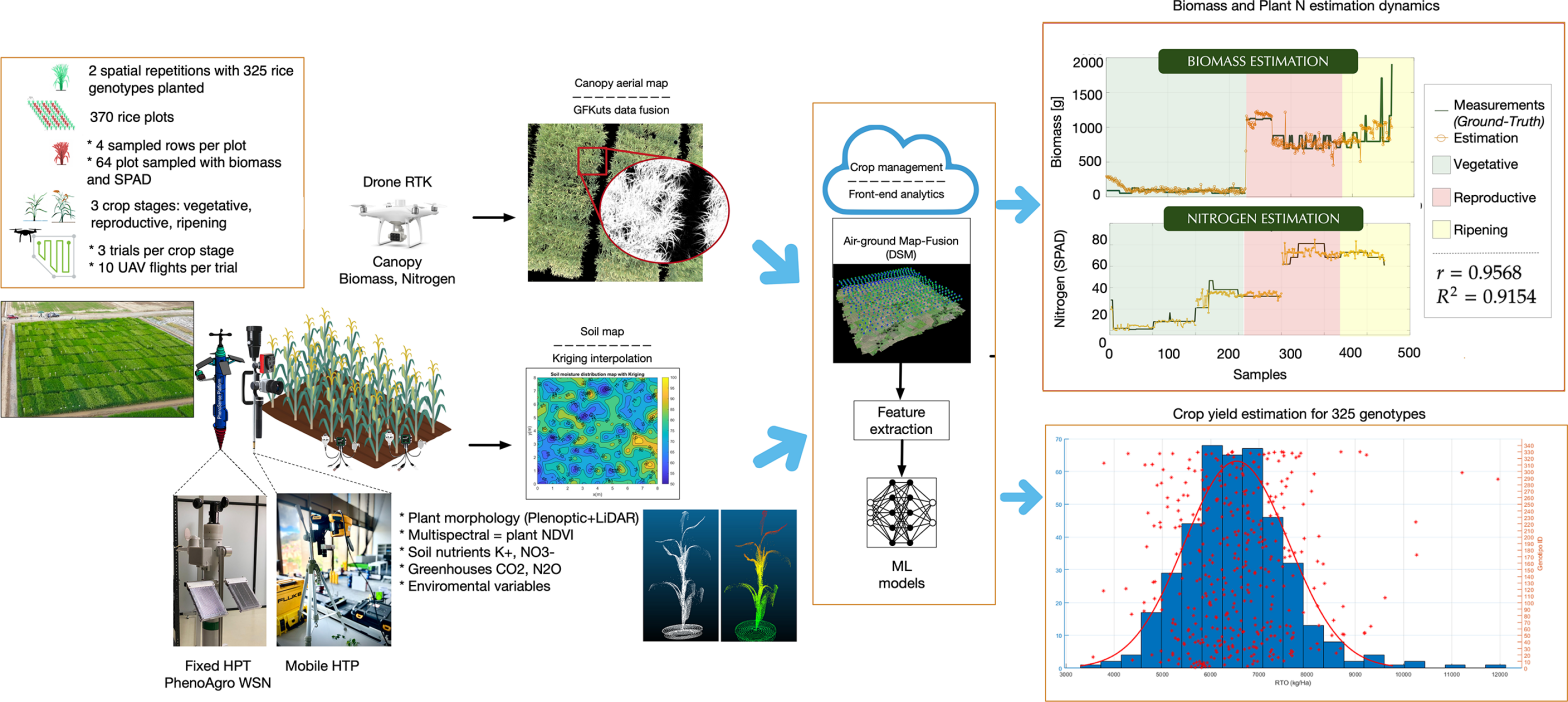
The OMICAS alliance has developed, validated and is currently deploying three High-Throughput Phenotyping (HTP) strategies and integrated platforms: aereal, terrestrial-fixed and terrestrial-mobile. From the aereal, drone-based, multispectral imaging platform we are now able to predict leaf-N, NDVI, and other crop data; from the fixed ground-based systems we obtain soil-based nutrients, plant indices (including metabolite profiles) and atmospheric state variables (including greenhouse gas footprints from crops, primarily N_2_O and CH_4_).

In P4 we are developing a new HTP platform capable of measuring in real-time, among other variables: (i) soil nutrients (*K*+, *NO*3-) and gases (*CO*2, *N*2*O*), (ii) vegetative indices from individual plant architecture models, and (iii) above-ground biomass (AGB) and leaf nitrogen (*N*) estimation at crop canopy level (See **Figure 8**. At the ground-level, a central unit called PhenoAgro, integrates communication through a peer-to-peer wireless LoRa network, wifi communications to data-collecting and processing servers in a cloud configuration, and custom-designed sensors to determine the spatio-temporal evolution of ground, plant, and atmospheric variables. Furthermore, we have own developed our own image-processing and trajectory-control algorithms for commercial unmanned aerial vehicles (UAV), to estimate AGB and *N* content from canopy-level multispectral imagery.

We have demostrated the UAV platform in spatio-temporal characterization of different morphological and physiological variables in rice crops, specifically leaf nitrogen (*N*) and biomass production, both of which are good predictors of crop yield and plant health. Our above-ground methods to capture canopy traits, overcome the limitations of traditional destructive methods for biomass sampling, or the use of time-demanding soil plant analysis development chlorophyll meters (SPAD) for the estimation of leaf *N*. Most of the existing body of work uses multispectral aerial images for the calculation of canopy light reflectances at different wavelengths (Mishra et al., 2017; Yue et al., 2019; Maimaitijiang et al., 2019; Zhang et al., 2019b). Several features can be extracted from the aerial imagery to calculate vegetation index (VI) formulas, by associating specific reflectance bandwidths that are highly related to variations in leaf chemical components, leading to a proper estimation of biomass dynamics (Liu et al., 2017; Zhang et al., 2019a) and leaf *N* (Sun et al., 2017; Nigon et al., 2020).

Our UAV systems are equipped with a multispectral sensors (depicted in **Figure 8** to capture canopy imagery in the red, green, near-infrared (NIR) and red-edge bands. Images are collected through the entire phenological cycle of the crop, specifically during the vegetative, reproductive, and ripening stages of plant growth. In previous work (Correa et al., 2020), we presented a novel multispectral image segmentation method called GFKuts. The acquired aerial imagery is segmented by optimizing an energy fitness function that enables the proper labeling of texture in the red, green, and near-infrared space (RGN). The resultant RGN image-mask only includes pixel information that accurately represents the vegetation canopy, allowing for the proper extraction of VI-based features.

Several VIs have been proposed to associate specific spectral wavelengths with different crop variables (Lu et al., 2020). Nonetheless, no single set of VIs had been demonstrated across all crop stages and plant varieties, until our recent work (Devia et al., 2019), which identified and characterized a set of VIs suitable for the estimation of both AGB and leaf *N*, namely:

- Normalized Difference Vegetation Index (Kanke et al., 2016)
- Green Normalized Difference Vegetation Index (Prabhakara et al., 2015)
- Difference Vegetation Index (Naito et al., 2017)
- Corrected Transformed Vegetation Index (Naito et al., 2017)
- Soil-Adjusted Vegetation Index (Arroyo et al., 2017)
- Modified SAVI (Gnyp et al., 2014)
- Simple Ratio (Kanke et al., 2016)

The selected VIs exhibit a strong dependence on the NIR reflectance due to leaf chlorophyll absorption, providing an accurate approach for training machine learning models to estimate the accumulated canopy biomass and leaf nitrogen at each crop stage. **Figure 8** shows estimation results reported in (Colorado et al., 2020a; Colorado et al., 2020b). Artificial Neural Networks (ANN) are trained with the selected VIs to predict both AGB dynamics and N-to-SPAD correlations during the entire crop phenological stages. Correlations are obtained by comparing the estimations against an assembled ground-truth dataset with biomass and SPAD readings directly measured at ground-level. On average, we have obtained biomass correlations of *r*=0.9568 with *R* ^2^=0.9154, whereas *r*=0.986 with *R* ^2^=0.97 for leaf nitrogen. These are promising results towards the autonomous estimation of rice canopy AGB and N, with the aim of enabling high-resolution genome trait mapping for genomic selection models for plant improvement against abiotic stresses.

### 3.5 In-silico strategies for improved crop breeding

**Figure 9:**
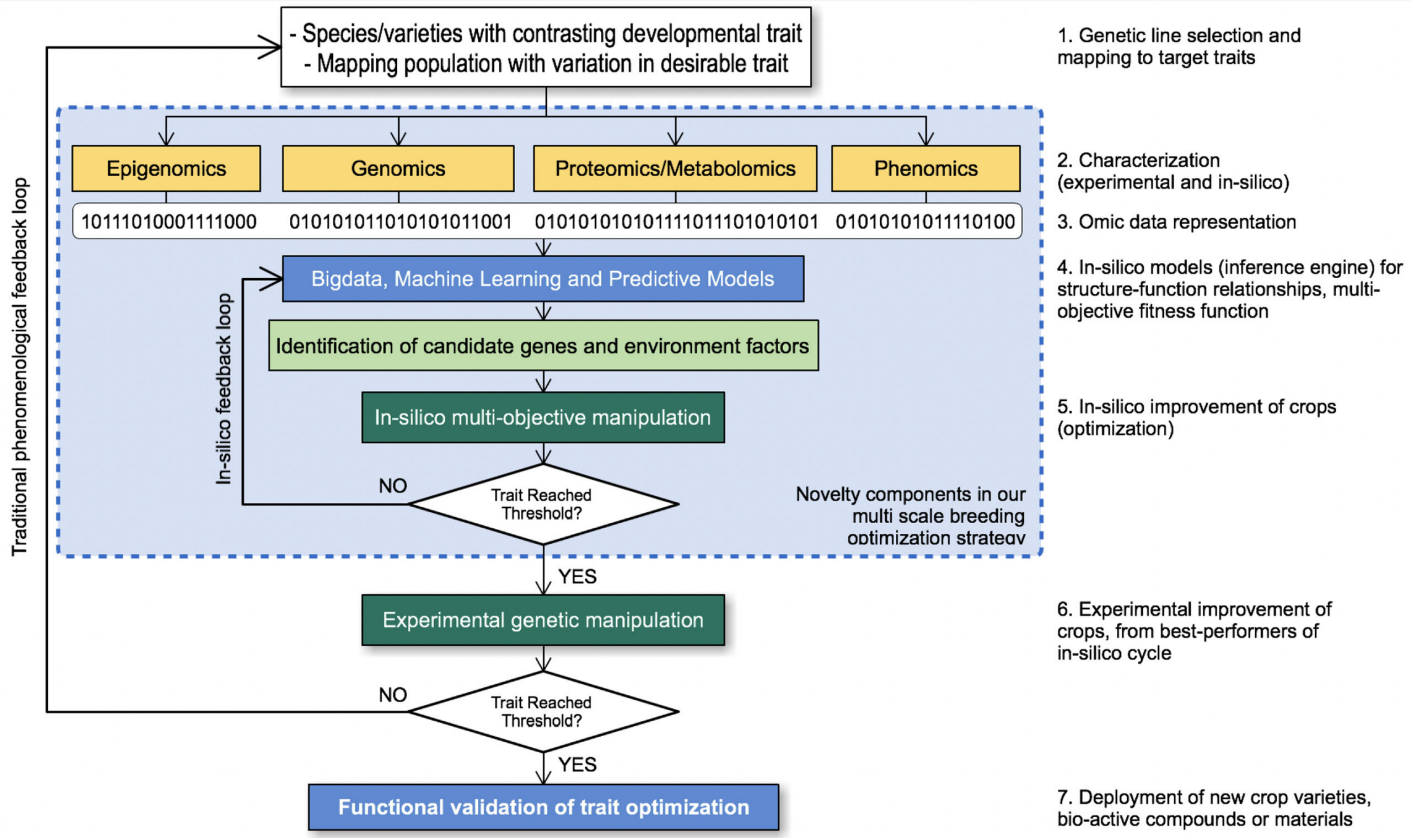
In-silico characterization strategy in OMICAS.

Plant breeding efforts generally require intensive labor as well as long optimization cycles that can last up to 12 years. **Figure 9** illustrates how the in-silico approach strategy in the OMICAS program accelerates traditional approaches, by narrowing down potential candidate species from a large set, based on fitness functions associated to one or more agronomic trait. This reduces the time and cost of experimental breeding and selection. Different components that may complement traditional approaches to plant breeding are grouped together inside the blue box in **Figure 9**. They take into account omic data representations, mathematical models and optimization algorithms to facilitate the identification of critical features that are present in populations with one (or more) desirable traits. Our goal in OMICAS has been to apply big data and machine learning algorithms on omics data characterized over multiple scales, in order to explore and ultimately uncover the key variables that intervene in stress-response and productivity. For example, an in silico approach may implement a computational environment to simulate critical optimization routes and explore a more ample and complete state/search space at a fraction of the time and cost. In P5, genetic, metabolic, protein, and cellular networks are used to supplement phenotypic traits associated to stress response, and to understand complex interactions and correlations upon which predictions can be based.

Our in-silico approach in OMICAS builds on epigenetic, genetic, metabolic and cellular regulation network models, characterized *via* results from P1-P4 to elucidate some of these complex interactions and correlations. Data analytics algorithms are used to identify and annotate genes associated to phenotypic traits. This in silico optimization cycle reduces the time and costs to breeding optimized agricultural plant varieties. It offers a significant advantages over the traditional labor-intensive scheme, among them:

- Discovering hidden relationships in large collections of data associated with crop productivity traits,
- Understanding the processes underlying the formation of these patterns,
- Quantifying productivity gain traits and their determinants, and
- Minimizing the genetic mutation and crossover space to optimize traits.

One particular path we have taken, addresses a common challenge in deploying new crop varieties, namely gene annotations and correlations. A variety of approaches to identify gene function/s have been proposed over the past years, including Weighted Gene Co-expression Network Analysis (WGCNA) (Langfelder and Horvath, 2008; Wang et al., 2020; Riccio et al., 2020). In (Riccio et al., 2020), for example, we proposed both a generalization and an extension of the original WGCNA, which is applied to rice (*Oryza sativa*. The proposed in silico approach identifies a group of 19 genes which are relevant in the response to salt stress. Such genes are considered target genes for experimental efforts to improve salinity tolerance in rice.

To identify the target genes, the approach relies on the idea of defining specific overlapping network ‘communities’ of genes, which are assumed to underlie the co-expression gene network. In other words, a key hypothesis of the proposed approach is that the overlapping nature of the systems’ regulatory domains that generate co-expression can be identified by applying an algorithm that detects modules of overlapping network communities. More specifically, module detection is achieved by using machine learning techniques of hierarchical link clustering. To analyze the phenotypic responses of each gene modules to salt stress, statistical regression analysis based on least absolute shrinkage and selection operator (LASSO) is employed. It is interesting to note that the identified target group is distributed across six classes: three that group together three genes associated to shoot K content; two that group three genes associated to shoot biomass; and finally, there one that groups four genes associated to root biomass. The proposed approach offers a framework to reduce the search-space for target genes that respond to salt stress. It facilitates experimental validation by reducing the number of relevant genes.

Leveraging on the tools, methods and technologies developed in P1 through P5, P6 focuses on optimizing crops through accurate and high-throughput phenotyping, gene and quantitative trait loci (QTL) discovery, molecular marker-assisted elite lines construction *via*, genomic selection (GS) and QTL-based marker-aided selection (MAS). P6 focuses on traits of high importance for the Colombian agricultural sector, and validates these on rice and sugarcane models. The traits are: (1) a biotic stress: *Rice hoja blanca virus* (RHBV) resistance, (2) three abiotic stresses: low radiation, high night temperatures and aluminum toxicity in rice, and (3) two physiological traits: sugar accumulation and nitrogen efficiency in sugarcane. These traits are highly relevant to crops in the region (e.g. RHBV resistance) and to crops worldwide (i.e. abiotic stresses and physiological traits).

### 3.6 Tolerance to Rice Hoja Blanca virus

**Figure 10:**
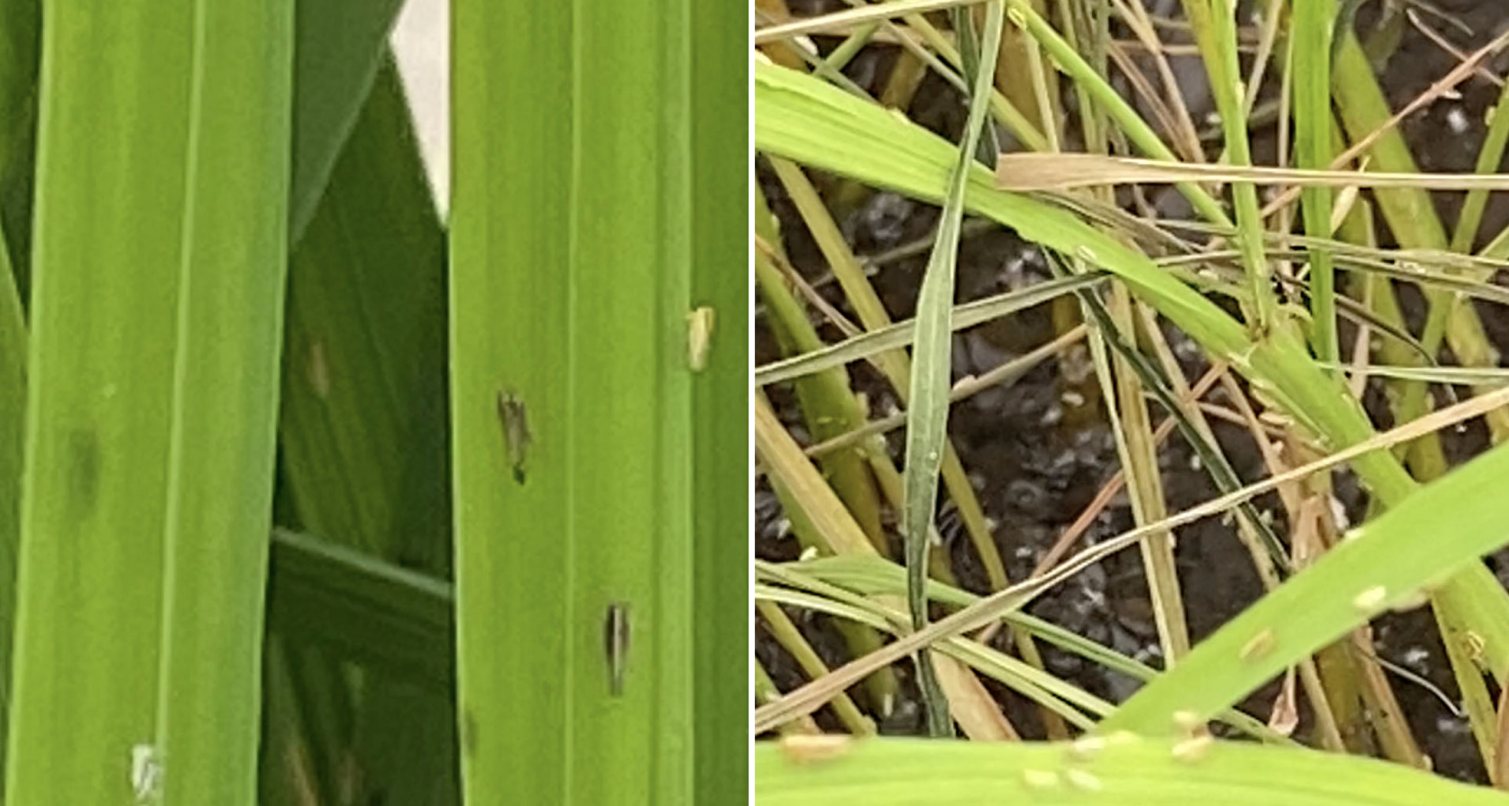
(left) Infected rice panicle showing planthopper insect and larvae, which transmits RHBV, and (right) physically damaged rice leaves.

The rice hoja blanca (RHB) disease is due to the *Rice hoja blanca virus* (RHBV), which is transmitted by a planthopper insect (see **Figure 10**, *Tagasodes oryzicolus*. RHB is among the most severe impediments to rice productivity in tropical Americas and the Caribbean Islands (Morales and Jennings, 2010). In Colombia it is the second threat for rice production after rice blast. There is no chemical or biological treatment available to fight the RHB disease, apart from devastating insecticides against its vector. Thus, tapping into diversity of genetic resistance to RHBV is key for a durable, successful, environment- and consumers health-friendly, integrated crop management. In P6 we take advantage of an extensive screening of rice germplasm to map QTLs that control the incidence and the severity of the disease in four diverse resistance donors selected amongst the best performers against the disease (Cruz-Gallego et al., 2018). We also search for possible interactions (epistasis) between the QTLs. And finally we identify candidate genes underlying the QTLs and attempt to validate them using a CRISPR-Cas9-based knock-out approach. The knowledge produced on genes and QTL represents the basis for a modern approach of marker-aided breeding of elite rice lines resistant to RHBV.

Our main result so far is the discovery of a major QTL for resistance to the virus in two Colombian cultivars, FD 50 and FD 2000, as well as two QTLs that control the damages caused by the insect vector (Romero et al., 2014). This QTL for RHBV resistance, renamed as *qHBV4.1*, controls RHBV *incidence*, which is simply the percentage of plants that show symptoms of viral infection, no matter the extent or severity of the symptoms. A local ancestry analysis in the *qHBV4.1* region showed that both resistant cultivars share the same temperate Japonica origin, although FD 50 and FD 2000 are mostly Indica germplasm. Thus, there is a high risk of resistance breakdown by a mutation in the virus RNA, due to a very poor diversity in resistant alleles in the cultivated germplasm. Recent observations of RHBV outbreaks even in the resistant FD 2000 near Cúcuta (Colombia) in FEDEARROZ plots tend to confirm the imminence of the threat. Other QTLs have been discovered by Genome-Wide Association Study (GWAS) but still need to be confirmed by bi-parental mapping (Cruz-Gallego et al., 2018). Incidence is the primary parameter to look at for the epidemics of a disease. Yet, its *severity* is certainly as important as incidence: if severity is low, a high incidence might have no significant impact on plant viability, panicle development, or grain yield. Additional to RHBV incidence, we thus designed new experiments to decipher the genetic control of RHBV resistance seen as symptoms severity. Through meta-QTL analysis using MapDisto v2 (Lorieux 2012; Heffelfinger et al., 2017) we found a new QTL, *qHBV11.1*, that controls HBV severity in three of the four crosses analyzed. Looking at the rice genome annotation (RAP-DB IRGSP v1) we also found an interesting candidate gene in the QTL region. This gene, STV11, was found to bring resistance to a cousin virus, the *Rice stripe virus* (Wang et al., 2014). We are currently investigating if STV11 in underlying the *qHBV11.1* QTL using *stv11* mutants created by CRISPR-Cas9 knock-out.

Using joint- and meta-QTL approaches, we could refine the *qHBV4.1* position. In the narrowed region of *qHBV4.1* we found a putative gene that encodes for the AGO-4 Argonaute protein (LOC-Os04g06770). Argonaute proteins, in addition to participating in the regulation of endogenous gene expression, also play a critical role in the defense against viruses through small interference RNA of viral origin which bind to Argonaute and serve as a guide for it to cut new viral RNA particles (Mallory and Vaucheret, 2010). This system is a common defense mechanism against pathogens, so AGO-4 may also be associated with resistance to RHBV. We investigated the action of *qHBV4.1* using *stv11* mutants created by CRISPR-Cas9 knock-out in the resistant genetic background FD-2000. We found a mutant that showed higher RHB incidence than the wild FD-2000, indicating that AGO-4 is a major factor of resistance to RHBV.

### 3.7 Low radiation and high noctural temperature tolerance in rice crops

Studies on climate variability impact on rice yield showed that low radiation is an important yield limiting factor (Sheehy et al., 2006; Yang et al., 2014). Low radiation constrained yield with about 40 to 50% yield loss in rice grown in India and south east asia countries (Viji et al., 1997; Sekhar et al., 2019) and Colombia (Delerce et al., 2016). Furthermore, the lack of optimal windows for farmers to sow either due to climatic or management constrains increases the probability of rice crops to find low radiation conditions at the end of the cycle. Plant traits related to source: sink interaction as carbon partition to grains rather than plant traits related to source activity (photosynthesis) are related to rice plant tolerance to low radiation during grain filling conditions (Shao et al., 2019; Shao et al., 2021). Our project, will look for traits and genes that allow to discriminate low radiation tolerant plants. Field phenotyping in two sites in Colombia of two populations (MAGIC indica and Diversity indica panel) and GBS genotyping will allow us to find out stable QTLs confering tolerance to low radiation to rice.

Despite being a mainly tropical crop, rice is heat sensitive but avoids daytime heat stress *via* transpirational cooling. This works less well at night, resulting in yield reductions through reduced grain filling rates and duration, smaller endosperm cell number and loosely stacked starch. The latter causes chalkiness, an important parameter for grain quality. These High Night Temperature (HNT) effects result from impeded grain development (sink formation) and ‘starvation’ (source limitation). Possibly, massive increases in panicle respiration are insufficiently offset by increased photosynthesis and reserve mobilization. Evidence is building that HNT causes significant yield reductions in some tropical environments, and this is expected to be aggravated by global warming: Night temperatures rise faster than day temperatures (Davy et al., 2017). Late-season HNT caused 40% yield loss locally in LA and the Caribbean (Delerce et al., 2016) and Asia (Welch et al., 2010). Tolerance to HNT is uncommon among high-yielding rices and has not been explicitly bred for. New phenotyping platforms and the search for traits, tolerant genotypes and favorable alleles in heat stress responsive genes during OMICAS will provide opportunities to develop cultivars tolerant to heat. Tolerant varieties as N22, the most heat tolerant genotype known (Jagadish et al., 2015), is a poor trait donor due to undesirable agronomic traits and genetic distance to the genetic background of high-yielding cultivars. New sources of tolerance are needed. Donors for HNT tolerance may also be sought in distant genomes. A major QTL for thermotolerance was identified and cloned in African rice (Oryza glaberrima). Thermo-tolerance 1 (TT1) encodes an *α*2 subunit of the 26S proteasome involved in the degradation of ubiquitinated proteins. The OgTT1 allele of heat-tolerant cv. CG14, expressed in a sensitive cultivar, eliminated cytotoxic denatured proteins. Overexpression of OgTT1 was associated with enhanced thermotolerance in rice, A. thaliana and Festuca (Li et al., 2015), although effects on grain quality remain unknown. Some genetic variation for HNT tolerance exists within sativa rice. Some QTLs and genes associated with HNT tolerance were characterized but have not yet been field-validated and introduced to breeding (Janni et al., 2020). More multidisciplinary translational research is needed to develop high-yielding varieties adapted to HNT tolerance and climate change. The grain filling stage in rice is the most sensitive stage to a reduction in radiation. From a physiological angle low radiation can reduce the source of carbon (Wang et al., 2015) but can also affect sink number and activity (Cantagallo et al., 2004). The impact on sink, has not been demonstrated in rice and deserves further studies. The difficulty to find a relevant trait related to low radiation tolerance during grain filling in previous studies generates also on the lack of genetic diversity studied and the difficulty to phenotype large and diverse panels for a stress during a specific phonological phase (Wang et al., 2015).

For low radiation tolerance during grain filling, we carried out phenotype-genotype evaluation on a rice diversity panel using whole genome association studies (GWAS). An Indica panel (300 accessions) was evaluated in the field, during two consecutive years. Grain yield, fertility, 1000 Grain weight, stem-leaf ratio, source:sink relation (SSR) and the number of filled grains per panicle were significantly reduced by low radiation and significantly different across genotypes. A total of 108 QTLs (Quantitative trait loci) with a log 10^-4^ significance, were associated with 20 variables evaluated in the high and low radiation treatments. For low radiation conditions, two common QTLs were found. The OSGRAS19 gene associated in previous studies with grain size (Sink size) and light interception (Source activity) was identified in the LD region of the QTL associated for a proxy trait measured in plants as an indicator of the ability of the plant source organs to fill the grains. A validation of the candidate gene in a MAGIC indica population and the introgration into elite breeding material is ongoing. Concurrently, we will perform a functional analysis of the QTL using tools from P1. During the first two years of the project we have evaluated a set of 30 heat temperature genotypes in a hot spot for hight temperatures in Colombia (Saldaña). However, due to la Niña, we only got one year with real HNT. Hot spot sites are relevant to screen materials, however the stresses are difficult to control and need multi-year trials to find out trend similarities. In order to impose HNT treatment at key developmental stages, we installed controlled heat tents at CIAT to maintain an elevated temperature only through the night. Currently, we are evaluating 140 genotypes (with the 30 genotypes evaluated at Saldaña). This evaluation will allow us to validate the platform for HNT and to suggest candidate parental lines to the breeding program. Along with P4, we are adapting phenotypic tools to continuously sense plants temperature at night within this platform.

### 3.8 Al^3+^ plant toxicity in acid soils

High concentrations of free aluminum (*Al* ^3+^) and drought are the main constraints to rice productivity in the Llanos Orientales of Colombia, the most important rice-producing area in the country, in terms of extension. The genetic improvement of drought and aluminum toxicity tolerance has been intensively studied worldwide, with major advances in identifying the genes that regulate these responses and even with the release of rice varieties with high tolerance to these stresses. However, few studies address the impact of the simultaneous occurrence of drought and aluminum toxicity on rice yield, and even fewer focus on identifying the best gene/allele combinations to increase tolerance to these stresses. In the Omicas program, we study these stresses by focusing on: 1) identifying genes/alleles associated with increased cross-tolerance, 2) establishing the regulatory mechanisms, including epigenetics, that determine the difference in tolerance between rice genotypes, and 3) supporting the release of rice varieties adapted to the conditions of the Llanos Orientales of Colombia. For the discovery of genes and allelic variants, we are exploiting the robustness of a synthetic population developed by CIRAD and CIAT for the upland rice-breeding program for Latin America and the Caribbean. Gene discovery is based on GBS-GWAS over the entire synthetic population (some 300 lines). To accelerate the release of tolerant rice varieties, we have selected advanced lines from this synthetic population, which have already been evaluated under the environmental conditions prevailing in the Llanos Orientales and exhibited variability in grain yield and root system characteristics. These advanced lines will be used to identify allelic variants through targeted sequencing of genes with a major influence on the response to aluminum and drought (*Nrat1, STAR1, STAR2, FRDL4, ARS5, ART1, Dro1*, qQTY 2.2, 4.1). If stable favorable haplotypes are identified through these approaches, molecular markers will be developed for use in marker-assisted selection (MAS) and introgression into elite varieties *via* marker-assisted backcrossing (MABC). Epigenetic regulation will be evaluated by sequencing the methylomes of genotypes with contrasting tolerance levels.

### 3.9 Greenhouse gas emissions from agricultural crops

Nitrogen comprises 78% of the earth’s atmosphere and its oxides (nitrous and nitric oxide, *N*2*0* and *NO*, respectively) play an important role in the biogeochemical cycle of *N* but its emission from the ground also has a great environmental impact. Nitrous oxide is not only a powerful greenhouse gas, it is the most depleting substance in stratospheric ozone. According to the Fifth Assessment Report of the Intergovernmental Panel on Climate Change (IPCC, 2013), cultivated soils and natural vegetation contribute 5.0–13.8 Tg *N*2*O*-*N* annually (Yao et al, 2020). In soils, *N*2*O* emissions are closely linked to the microbial processes of nitrification and denitrification, but nitrification rarely produces more than 1% of the *N*2*O* emission from agricultural soils, leaving denitrification, especially in soils with high moisture content, as the major source of *N*2*O* in agricultural soils (SYL, 1999). The knowledge of the emission rate from different agricultural production systems will allow to fine-tune their management in order to minimize the emission of *N*2*O*.

Brazil, followed by Australia, are the countries where the largest number of studies have been carried out to evaluate the emission of *N*2*O* from sugarcane. In other countries of Central and South America, including Colombia, these studies have been very scarce (Valencia et al, in preparation). In recent years, according to the Third National Communication on Climate Change, Colombia went from emitting 0.37% of global emissions to 0.46% (11 metric tons of *CO*2), ranking 5^th^ out of32 countries in Latin America and the Caribbean. Sugarcane is one of the most important agro-industrial crops in Colombia, but unfortunately there is not enough information about the emission of GHG from this production system, so our work in the Omicas program will provide a first quantitative view of *N*2*O* emissions from this crop under agroindustrial production conditions in the Cauca river valley.

In P7, we implemented a study to quantify the emission of *N*2*O* in fields of commercial sugarcane production in two contrasting environments in terms of soil moisture, humid environment, where evapotranspiration is less than precipitation, and a dry environment, where evapotranspiration is greater than precipitation. Our preliminary results show that, in consistency with similar studies in other countries, soil moisture and nitrogen fertilization are the main factors that determine the intensity of *N*2*O* emission. After nitrogen fertilization, an increase in the emission of *N*2*O* is observed, while the emission decreases over time, after fertilization increases.

### 3.10 Convergence and future prospects

The OMICAS program brings a trans-disciplinary approach into crops breeding. It couples theory, lab, field and computational experiments within a multiscale omics characterization strategy that enables breeding and validation of new varieties with improved agronomical traits; with higher precision, and in a cost-effective and timely manner. Within three years of its launch, the OMICAS team has, among others:

- uncovered epigenetic differences from four commercial rice cultivars and two accessions of wild rice associated to *Al* ^3+^ toxicity tolerance,
- developed and validated novel graph-theory and machine learning tools to annotate genes using topological properties from co-expression networks, Identified 13 SNP biomarkers and 20 candidate genes associated to sucrose production in sucarcane,
- designed, developed and validated new nano-sensors for the detection and quantification of biomarkers (primary and secondary metabolites) related to a plant organism’s health, heavy metals (*Al* ^3+^) that compromise nutrient absorption, and gases (methane and nitrous oxide) that contribute to the greenhouse footprint of agriculture,
- predicted the tertiary structure of a key membrane protein for stress signaling in plants, from which we are currently studying the plausible signaling pathways through two plant hormones (Abscisic acid [ABA] and gibberellins [GA1]),
- designed, developed and validated a high-throughput phenotyping platform that integrates real-time data from fixed, mobile terrestrial with aerial devices to characterize soil, plant, atmosphere and crop variables,
- identified different rice genes that confer tolerance to RHBV, high nocturnal temperatures, low-radiation, and to aluminum toxicity,
- applied gene-editing technologies (mainly site-specific nuclease (SSN) with CRISPR/Cas) to produce experimental rice crops with enhanced stress response to RHBV, and improved resource use efficiency (Nitrogen and water) and higher yields for both rice and sugarcane, and
- quantified and mapped the emission of nitrous oxide, methane and carbon dioxide from commercial sugarcane production in contrasting environments in Colombia.

We expect the OMICAS strategy, methods and tools will continue to have an incremental impact on breeding of new varieties, beyond rice and sugarcane, and on general agricultural practices. As epigenome and genome-wide characterizations studies lead to function discovery, and our understanding of stress signaling pathways and identification of response mechanisms progresses, we expect a move from editing single or a few nucleotides, to full allele replacement, and ultimately new functional gene insertions.

Such a broad and in-depth characterization effort poses enormous challenges, in terms of the combinatorial explosion of datum, the inherent complexity of deep/hidden interrelationships, and of the non-deterministic nature of multi-objective optimizations will require new processing and interpretation capabilities that are discipline-agnostic. Notwithstanding, this strategy will lead, not only to crops that can resist pests and thrive in difficult climates, but to significant nutritional value improvements, all of which contributes to food security, sustainable productivity, and to the democratization of food production, which disproportionately affects the poorest and most vulnerable people today.

## Data availability statement

The original contributions presented in the study are included in the article/Supplementary Material. Further inquiries can be directed to the corresponding author.

## Author contributions

All authors contributed to writing and revising the manuscript. AJ-B conceived the program’s multiscale strategy and integrated omics architecture. JC, LT, MC and AJ-B contributed directly to phenotyping platforms and sensors, MQ and KV contributed to plant epigenomics and genomics analysis, MR and TG-H to studies on rice abiotic stress tolerance, ML to RHBV stress tolerance, AJ-B, WG and CA to GCR1 structure prediction and function in stress tolerance, JF and CR to computational strategies for gene annotation, FM, NC and AJ-B to GHG characterization of crops, and FS and JR contributed to sugarcane genomic characterization. All authors contributed to the article and approved the submitted version.

## Funding

This work was partially funded by the “OMICAS program: Optimización Multiescala In-silico de Cultivos Agrícolas Sostenibles (Infraestructura y validación en Arroz y Caña de Azúcar)” Scientific Ecosystem belonging to the Scientific Colombia Program, sponsored by The World Bank, The Ministry of Science, Technology and Innovation (MinCiencias), ICETEX, the Colombian Ministry of Education and the Colombian Ministry of Commerce, Industry and Turism, under GRANT ID: FP44842-217-2018, Award ID: 792-61187.

## Acknowledgments

The authors would like to recognize all the students and technical staff sponsored by the program, from the different institutions involved, for their dedication and contributions to the work reported here, including: Juan M. Marmolejo, Jhonattan de la Roche, Mauricio Peñuela, Jenny Gallo, Chrystian Sosa, Gustavo Lara, Sammy Perdomo, Pedro M. Hernández, David Jimenez, Miguel A. Romero, Nicolás López, Camila Riccio, Sandra Loaiza, Manuel Valencia, Vanessa Reyes, and Juliana Chaura. The authors would also like to thank Dr. Edison Suarez, Ana Claudia Gordillo, and Sandra Losano from Minciencias, father Luis F. Gomez, S.J., Carlos Monthermoso, Ingrid Schuler, Jaime Aguilar, Leidi Rojas, Yaneth Rodríguez, Alejandra Catano, and Michael Hernández from the PUJC, and the alliance’s board members Joe Tohme from CIAT-CGIAR, Rodomiro Ortiz from the Swedish University of Agricultural Sciences, José H. Bahamón from Universidad Icesi, Jorge H. Victoria, Patricia Guzmán from Fedearroz, Juan Manuel Chaves from REDDI, and Marcela Arrivillaga from PUJC.

## Conflict of interest

The authors declare that the research was conducted in the absence of any commercial or financial relationships that could be construed as a potential conflict of interest.

## Publisher’s note

All claims expressed in this article are solely those of the authors and do not necessarily represent those of their affiliated organizations, or those of the publisher, the editors and the reviewers. Any product that may be evaluated in this article, or claim that may be made by its manufacturer, is not guaranteed or endorsed by the publisher.
