# Corrigendum: The ÓMICAS alliance, an international research program on multi-omics for crop breeding optimization

**DOI:** 10.3389/fpls.2022.1104501

**Published:** 2022-12-20

**Authors:** Andres Jaramillo-Botero, Julian Colorado, Mauricio Quimbaya, Maria Camila Rebolledo, Mathias Lorieux, Thaura Ghneim-Herrera, Carlos A. Arango, Luis E. Tobón, Jorge Finke, Camilo Rocha, Fernando Muñoz, John J. Riascos, Fernando Silva, Ngonidzashe Chirinda, Mario Caccamo, Klaas Vandepoele, William A. Goddard

**Affiliations:** ^1^ Chemistry and Chemical Engineering Division, California Institute of Technology, Pasadena, CA, United States; ^2^ Departamento de Ingeniería Electrónica, Facultad de Ingeniería, Pontificia Universidad Javeriana, Bogotá D.C., Colombia; ^3^ Departamento de Ciencias Naturales y Matemáticas, Facultad de Ingeniería y Ciencias, Pontificia Universidad Javeriana, Cali, Valle del Cauca, Colombia; ^4^ CIRAD, UMR AGAP, Montpellier, France; ^5^ AGAP, Univ Montpellier, CIRAD, INRA, Montpellier SupAgro, Montpellier, France; ^6^ International Center for Tropical Agriculture (CIAT), Cali, Valle del Cauca, Colombia; ^7^ DIADE, University of Montpellier, CIRAD, IRD, Montpellier, France; ^8^ Departamento de Ciencias Biológicas, Facultad de Ciencias Naturales, Universidad Icesi, Cali, Colombia; ^9^ Departamento de Ciencias Químicas, Facultad de Ciencias Naturales, Universidad Icesi, Cali, Valle del Cauca, Colombia; ^10^ Departamento de Ingeniería Electrónica y Ciencias de la Computación, Facultad de Ingeniería y Ciencias, Pontificia Universidad Javeriana, Cali, Valle del Cauca, Colombia; ^11^ Centro de Investigación de la Caña de Azúcar (CENICAÑA), Florida, Valle del Cauca, Colombia; ^12^ OMICAS Alliance, Pontificia Universidad Javeriana, Cali, Valle del Cauca, Colombia; ^13^ National Institute of Agricultural Botany (NIAB), Cambridge, United Kingdom; ^14^ Department of Plant Biotechnology and Bioinformatics, Ghent University, Gent, Belgium; ^15^ Center for Plant Systems Biology, Vlaams Instituut voor Biotechnologie (VIB), Gent, Belgium

**Keywords:** Multi-omics, crops breeding, foodomics, nanotechnology, rice and sugarcane, in-silico optimization

In the published article, there were errors in affiliations 2-14.

Instead of affiliation 2 being “Optimización Multiescala In-silico de Cultivos Agrícolas Sostenibles (OMICAS) Alliance, Pontificia Universidad Javeriana, Cali, Colombia”, it should be affiliation 12 and updated to “OMICAS Alliance, Pontificia Universidad Javeriana, Cali, Valle del Cauca, Colombia”.

Instead of affiliation 3 being “Facultad de Ingeniería y Ciencias, Departamento de Ciencias Naturales y Matemáticas, Pontificia Universidad Javeriana, Cali, Colombia”, it should be affiliation 2 and updated to “Departamento de Ciencias Naturales y Matemáticas, Facultad de Ingeniería y Ciencias, Pontificia Universidad Javeriana, Cali, Valle del Cauca, Colombia”.

Instead of “CIRAD, UMR AGAP, Montpellier, France” being affiliation 5, it should be affiliation 4.

Instead of “AGAP, Univ Montpellier, CIRAD, INRA, Montpellier SupAgro, Montpellier, France” being affiliation 6, it should be affiliation 5.

Instead of affiliation 7 being “International Center for Tropical Agriculture (CIAT), Cali, Colombia”, it should be affiliation 6 and updated to “International Center for Tropical Agriculture (CIAT), Cali, Valle del Cauca, Colombia”.

Instead of “DIADE, University of Montpellier, CIRAD, IRD, Montpellier, France” being affiliation 8, it should be affiliation 7.

Instead of affiliation 9 being “Facultad de Ciencias Naturales, Departamento de Ciencias Biológicas, Universidad Icesi, Cali, Colombia”, it should be affiliation 8 and updated to “Departamento de Ciencias Biológicas, Facultad de Ciencias Naturales, Universidad Icesi, Cali, Valle del Cauca, Colombia”.

Instead of affiliation 10 being “Facultad de Ciencias Naturales, Departamento de Ciencias Químicas, Universidad Icesi, Cali, Colombia”, it should be affiliation 9 and updated to “Departamento de Ciencias Químicas, Facultad de Ciencias Naturales, Universidad Icesi, Cali, Valle del Cauca, Colombia”.

Instead of affiliation 11 being “Facultad de Ingeniería y Ciencias, Departamento de Electrónica y Ciencias de la Computación, Pontificia Universidad Javeriana, Cali, Colombia”, it should be affiliation 10 and updated to “Departamento de Ingeniería Electrónica y Ciencias de la Computación, Facultad de Ingeniería y Ciencias, Pontificia Universidad Javeriana, Cali, Valle del Cauca, Colombia”

Instead of affiliation 12 being “Centro de Investigación de la Caña de Azúcar de Colombia, Centro de Investigación de la Caña de Azúcar (CENICAÑA), Cali, Colombia” it should be affiliation 11 and updated to “Centro de Investigación de la Caña de Azúcar (CENICAÑA), Florida, Valle del Cauca, Colombia”.

Instead of affiliation 13 being “National Institute of Agricultural Botanics (NIAB), Cambridge, United Kingdom” it should be “National Institute of Agricultural Botany (NIAB), Cambridge, United Kingdom,”.

Instead of affiliation 14 being “Vlaams Instituut voor Biotechnologie, Bioinformatics Systems Biology, Ghent University, Gent, Belgium”, it should be “Department of Plant Biotechnology and Bioinformatics, Ghent University, Ghent, Belgium”.

Instead of affiliations indices 1,2* for Andres Jaramillo-Botero, they should be 1,12*

Instead of affiliations indices 2,3 for Julian Colorado, they should be 2,12

Instead of affiliations indices 2,4 for Mauricio Quimbaya, they should be 3,12

Instead of affiliations indices 2,5,6,7 for Maria Camila Rebolledo, they should be 4,5,6,12

Instead of affiliations indices 2,7,8 for Mathias Lorieux, they should be 6,7,12

Instead of affiliations indices 2,9 for Thaura Ghneim-Herrera, they should be 8,12

Instead of affiliations indices 2,10 for Carlos A. Arango, they should be 9,12

Instead of affiliations indices 2,11 for Luis E. Tobón, they should be 10,12

Instead of affiliations indices 2,11 for Jorge Finke, they should be 10,12

Instead of affiliations indices 2,11 for Camilo Rocha, they should be 10,12

Instead of affiliations indices 2,12 for Fernando Muñoz, they should be 11,12

Instead of affiliations indices 11,14 for John J. Riascos, they should be 11,12

Instead of affiliations indices 2,12 for Fernando Silva, they should be 11,12

Instead of affiliations indices 2,7 for Ngonidzashe Chirinda, they should be 6,12

In the published article, there was an error regarding the affiliations for Klaas Vandepoele.

As well as having affiliation 14, he should also have “Center for Plant Systems Biology, Vlaams Instituut voor Biotechnologie (VIB), Ghent, Belgium”.

The authors apologize for these errors and state that this does not change the scientific content or conclusions of the article in any way. The original article has been updated.

